# Amelioration of psoriasis-like skin lesions by human amniotic mesenchymal stem cells: insights from multiomics profiling in mice

**DOI:** 10.3389/fimmu.2026.1776874

**Published:** 2026-02-17

**Authors:** Liehao Yang, Baihui Miao, Qian Sun, Fangqing Zhang, Hongyan Sun, Zilong Zhou, Yue Hu, Zhiming Cui, Dongxu Wang, Chenlu Liu, Ling Zhang, Qianying Hu, Xianling Cong

**Affiliations:** 1 Department of Dermatology, China-Japan Union Hospital of Jilin University, Changchun, China; 2 Department of Biobank, China-Japan Union Hospital of Jilin University, Changchun, China; 3 Institute of Antler Science and Product Technology, Changchun Sci-Tech University, Changchun, China; 4 Key Laboratory of Pathobiology, Ministry of Education, Department of Biomedical Science, College of Basic Medical Sciences, Jilin University, Changchun, China

**Keywords:** human amniotic mesenchymal stem cell, MMP9, psoriasis, S100A9, single cell

## Abstract

**Background:**

Psoriasis is a multifactorial, chronic inflammatory skin disease. Current treatment modalities are limited by suboptimal patient responses and high recurrence rates after discontinuation. Consequently, there is an urgent need to develop novel therapeutic strategies for psoriasis.

**Methods:**

An imiquimod‐induced mouse model of psoriasis was established, and human amniotic mesenchymal stem cell (hAMSC) were subsequently administered to evaluate their therapeutic efficacy. Bioinformatic analyses of Gene Expression Omnibus (GEO) datasets GSE39035 and GSE97311 were performed to identify potential hAMSC therapeutic target genes. Using data from GSE228421, a single-cell transcriptomic atlas of psoriasis was constructed to examine the distribution and functional roles of these target genes across different cell populations.

**Results:**

By integrating an imiquimod-induced murine model with comprehensive bioinformatic analyses of GEO datasets, we demonstrated that hAMSC administration significantly ameliorated psoriasis-like skin lesions, restored epidermal architecture, and reduced PASI and Baker scores. This therapeutic efficacy was accompanied by the alleviation of splenomegaly and a systemic reduction in inflammatory cytokines (IL-17 and TNF-α) without inducing hepatotoxicity. *In vitro* experiments further confirmed that hAMSCs inhibited TNF-α-induced keratinocyte proliferation and reactive oxygen species (ROS) generation. Transcriptomic profiling identified key immune-related targets, revealing that hAMSCs significantly modulated the expression of genes such as MMP9, S100A9, and BACH2. It is worth noting that single-cell atlas analysis has revealed that S100A9 and MMP9 play significant roles respectively in psoriasis-related CD8-IL17A T cells and M2-like macrophages, and further clarified the functional characteristics of S100A9 in the temporal development process of psoriasis fibroblasts and keratinocytes.

**Conclusions:**

In summary, our findings confirm the efficacy and safety of hAMSCs in the treatment of psoriasis and elucidate the underlying mechanisms of their therapeutic action.

## 1 Introduction

Psoriasis is a common, immune-mediated, chronic inflammatory skin disease that can occur at any age, with an average age of onset of 33 years (1). Psoriasis typically affects the skin, nails, and joints and is often associated with a number of comorbidities, including psoriatic arthritis and cardiovascular disease (1, 2). The pathophysiology of psoriasis is characterized by hyperproliferation and abnormal differentiation of keratinocytes, dilation and elongation of capillaries, and inflammatory infiltration of T cells in the dermis and epidermis (1, 3). Compared with the general population, people with psoriasis are more likely to present with depression and have suicidal tendencies (1, 4). The disease not only affects the quality of life of patients but also profoundly affects their mental health.

Psoriasis is a multifactorial disease in which genetics, immune system factors, and environmental exposures all contribute significantly, with immunological factors playing a particularly crucial role in its pathogenesis (5, 6). Key cytokines such as TNF-α, IL-17A, IL-17F, and IL-22 produced by T cells, especially activated Th1 and Th17 cells, further stimulate keratinocytes, which in turn release a variety of cytokines, chemokines, and antimicrobial peptides that sustain a proinflammatory response (7). The interaction between hyperproliferative epidermal keratinocytes and activated immune cells forms a positive feedback loop (8, 9). However, the precise mechanisms underlying this process remain incompletely understood and require further investigation. Among the various treatment options for psoriasis, biologics have emerged as among the most effective therapies. These drugs work by targeting and inhibiting TNF-α, IL-17, IL-12, or IL-23 (10). However, they are not universally effective in all patients, and there is a risk of recurrence, highlighting the urgent need for the development of new therapeutic strategies.

In recent years, mesenchymal stem cell (MSC) therapy has received considerable attention in the treatment of skin diseases (11). MSCs are a group of self-renewing pluripotent cells that can be isolated from a variety of sources, including bone marrow, umbilical cord blood, placenta, synovium and adipose tissue (12, 13). These cells play crucial roles in regulating immune responses through both autocrine and paracrine mechanisms and have shown promising therapeutic effects in cutaneous immune-related diseases such as psoriasis, lupus erythematosus, and pemphigus (11, 14). MSCs have been shown to exert anti-inflammatory effects by targeting key inflammatory mediators in inflammatory skin disorders and promoting the production of anti-inflammatory cytokines (15). In addition, several studies have reported the application of perinatal MSCs in human clinical trials. MSC-based therapies have achieved promising outcomes in the treatment of knee osteoarthritis, post-hip arthroplasty repair, atherosclerotic critical limb ischemia, and diabetic foot ulcers (16). Moreover, multiple studies have demonstrated the feasibility and therapeutic efficacy of umbilical cord-derived MSCs in the treatment of rheumatoid arthritis (16). Collectively, these findings highlight the broad clinical potential of MSCs in regenerative medicine and immune-mediated diseases.

Among perinatal tissue-derived MSCs, human amniotic mesenchymal stem cells (hAMSCs) have attracted increasing attention due to their unique biological characteristics. Compared with MSCs derived from other sources, hAMSCs exhibit extremely low immunogenicity, lack tumorigenic risk, do not raise ethical concerns, and possess robust proliferative capacity, potent anti-inflammatory properties, strong tissue repair potential, and pronounced paracrine activity (15, 17, 18). Accumulating evidence indicates that hAMSCs and their derived exosomes can effectively alleviate the severity of psoriatic skin lesions (19, 20). Based on these findings, we hypothesize that hAMSCs may represent a promising therapeutic strategy for psoriasis. However, the precise mechanisms by which hAMSCs exert their effects in psoriasis remain largely unexplored.

This study aimed to elucidate the effects and mechanisms of hAMSCs in the treatment of psoriasis. We identified immune-related differentially expressed genes to explore potential target genes of hAMSCs in psoriasis, among which S100A9 was an important target. By constructing a single-cell atlas, we explored the changes in this gene across different cell types in psoriatic tissues, thereby elucidating the role and mechanism by which human hAMSCs act on psoriasis by targeting S100A9.

## 2 Results

### 2.1 hAMSCs alleviate the skin phenotype in an imiquimod-induced psoriasis mouse model

**Figure 1:**
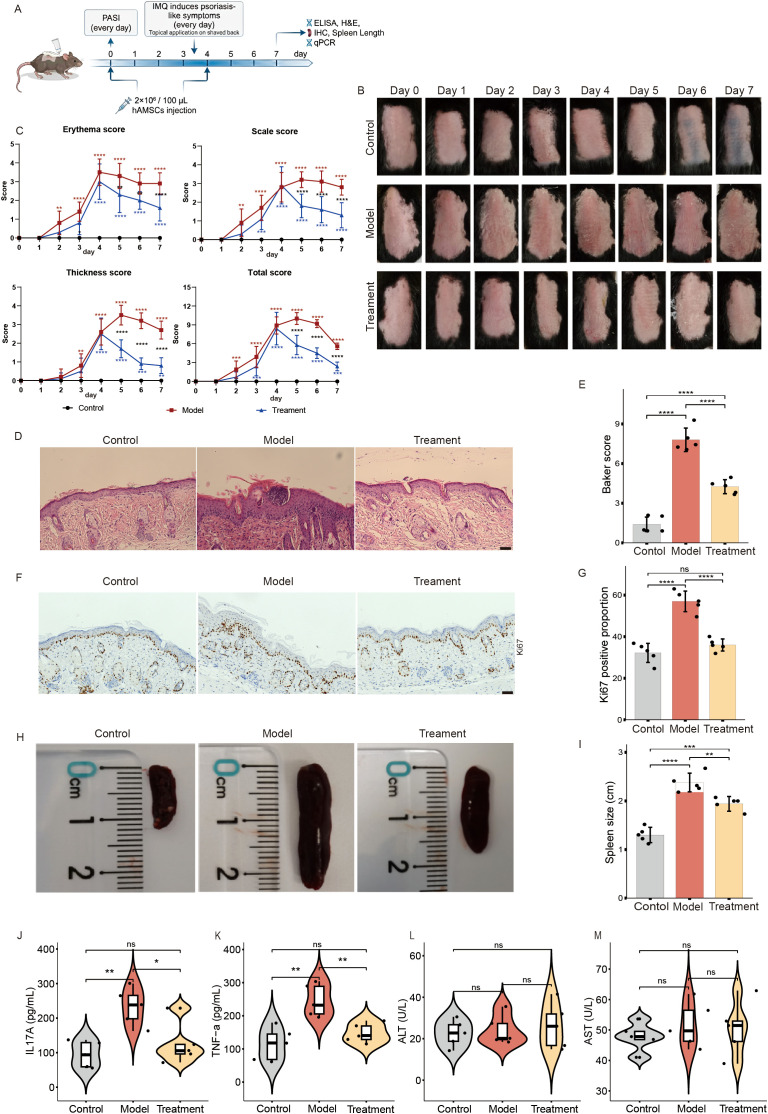
Effectiveness and safety of hAMSCs in psoriasis treatment. **(A)** Schematic representation of the experimental design. **(B)** Representative images of the dorsal skin of mice in the control, model, and treatment groups. **(C)** Progression of PASI scores (erythema, scale, thickness, and total score) in the three groups (control group, n=5; model/treatment group, n=10) from day 0 to day 7. Red * indicates statistical significance between the control and model groups; Blue * indicates statistical significance between the control and treatment groups; Black * indicates statistical significance between the treatment and model groups. **(D, E)** Representative H&E-stained skin section images, with statistical analysis performed using the Baker score (n=5) (Scale bars, 50μm). **(F, G)** Ki67 expression detected by immunofluorescence (IHC) (n=5) (Scale bars, 50μm). **(H, I)** Representative images of spleens from the three groups of mice, with quantitative analysis of spleen length (n=5). **(J, K)** The contents of IL17A and TNF-α in mice serum were detected using ELISA (n=5). **(L,M)** The contents of ALT and AST in mice serum were detected using ELISA (n=5). ns not significant, *P< 0.05, **P < 0.01, ***P < 0.001, ****P < 0.0001.

In this study, we investigated whether hAMSCs could be used for the treatment of psoriasis. To this end, we constructed a psoriasis mouse model using imiquimod cream (IMQ) (model group) and injected hAMSCs into the psoriasis mouse model on day 0 (1 day before modelling) and day 4 (treatment group) (**Figure 1A**). Compared with those in the normal group, the model group had obvious psoriasis-like skin lesions; however, after hAMSCs treatment on day 0, the severity of skin lesions in the psoriasis treatment group was significantly lower than that in the model group (**Figure 1B**), suggesting that hAMSCs also have a certain preventive effect on the occurrence of psoriasis. Furthermore, the decrease in the PASI score after the second administration of hAMSCs on the fourth day also confirmed that hAMSCs could significantly inhibit imiquimod induced psoriasis-like skin lesions in mice (**Figure 1C**). Histological analysis revealed that compared with the control group, the skin sections of the model group showed obvious pathological changes, including hyperkeratosis, parakeratosis, Munro microabscesses, and thickening of the spinous layer. hAMSCs could significantly improve the occurrence of such pathological changes (**Figure 1D**). Moreover, the Baker scores of the skin sections from the three groups of mice also yielded the same result (**Figure 1E**). Cellular proliferation marker Ki67 was decreased in the psoriasis model mice treated with hAMSCs as indicated by immunohistochemistry (**Figures 1F, G**). These results indicate that hAMSCs can alleviate the severity of psoriasis-like skin lesions and reduce epidermal hyperproliferation in psoriasis model mice.

To evaluate the effects of hAMSCs on inflammatory factors in psoriasis, we measured the serum levels of IL-17 and TNF-α in the three groups of mice. We found that hAMSCs significantly alleviated imiquimod-induced splenomegaly (**Figures 1H, I**) and the levels of inflammatory factors in the serum (**Figures 1J, K**). These results suggest that hAMSCs may play important roles in regulating inflammatory factor levels and modulating immune responses in psoriasis mice. In addition, to evaluate the safety of hAMSCs *in vivo*, we measured the serum alanine aminotransferase (ALT) and aspartate aminotransferase (AST) levels in the mice from the three groups (**Figures 1L, M**). We found no statistically significant differences among the groups, confirming the safety of hAMSCs for therapeutic application.

In addition, we established an *in vitro* psoriasis cell model using TNFα-stimulated HaCaT cells. The results showed that TNFα stimulation significantly enhanced the generation of reactive oxygen species (ROS) (**Supplementary Figures 1A, B**) and the proliferation ability (**Supplementary Figure 1C, D**) of HaCaT cells; while co-culture with hAMSCs significantly alleviated the biological effects induced by TNFα. This result suggests that hAMSCs may play a regulatory role in the *in vitro* psoriasis model by inhibiting cell proliferation and ROS generation. In conclusion, hAMSCs have demonstrated excellent therapeutic effects in both *in vivo* and *in vitro* models of psoriasis.

### 2.2 Screening of target genes affected by hAMSCs

**Figure 2:**
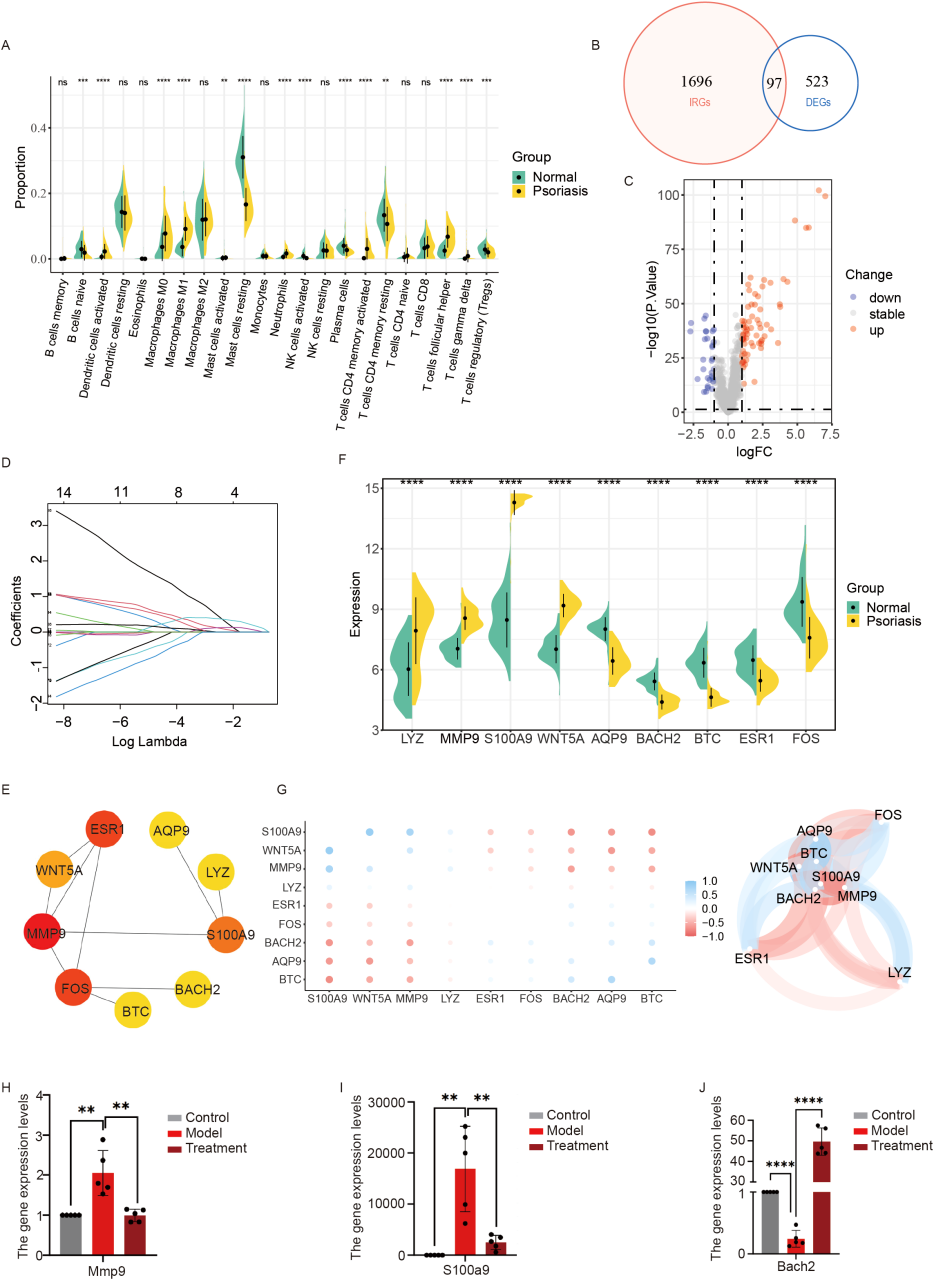
Screening of genes associated with hAMSCs for the treatment of psoriasis. **(A)** Differences in immune cell populations between normal and psoriatic tissues were assessed using the CIBERSORT algorithm applied to the GSE13355 and GSE14905 datasets. **(B)** Venn diagram showing the intersection of immune-related genes and DEGs (|logFC|>1, P value <0.05). **(C)** Volcano plot of immune-related DEGs. Blue nodes represent down-regulated genes in psoriasis, red nodes represent up-regulated genes, and gray nodes represent genes with no significant difference compared to normal tissues. **(D)** Identification of key psoriasis-associated immune-related genes using the LASSO logistic regression algorithm, with penalty parameter tuning conducted via 10-fold cross-validation. **(E)** Protein-protein interaction (PPI) network of key psoriasis immune-related differential genes (PIRGs). **(F)** Relative expression levels of hub PIRGs from the GSE13355 and GSE14905 datasets. **(G)** Heatmap and network diagram showing the correlation of expression among the 9 hub PIRGs. **(H)** Quantitative real-time PCR analysis of mRNA levels of Mmp9, S100a9 **(I)**, and Bach2 **(J)**, with statistical analysis presented (n = 5). Each experiment was repeated at least three times. ns not significant, **P < 0.01, ***P < 0.001, ****P < 0.0001.

To elucidate the pathogenesis of psoriasis and the mechanisms underlying the therapeutic effects of hAMSCs in psoriasis we measured the serum levels of inflammatory factors. We found that hAMSCs significantly suppressed these levels and also reduced spleen enlargement in psoriasis-like mice. On the basis of these findings, we hypothesized that hAMSCs may exert their effects by modulating immune-related functions in these mice. First, we predicted the abundance of infiltrating immune cells in psoriasis tissues using the CIBERSORT and ssGSEA algorithms applied to the microarray data. We found differences in the levels of various T cells (such as CD4 memory-activated T cells, regulatory T cells, and activated CD4 T cells), B cells (such as naïve B cells, activated B cells, and memory B cells), and mast cells (activated mast cells) in psoriasis tissue (**Figure 2A**; **Supplementary Figure 2A**). These findings suggest significant immune dysregulation in psoriasis tissue compared with normal tissue. Next, we intersected the differentially expressed genes (DEGs) identified in the psoriasis microarray data with the immune-related dataset from the ImmPort database (21), resulting in 97 psoriasis immune-related differential genes (PIRGs) (**Figure 2B**), including 67 upregulated genes and 30 downregulated genes (**Figure 2C**; **Supplementary Figure 2B**). We performed Gene Ontology (GO) and Kyoto Encyclopedia of Genes and Genomes (KEGG) enrichment analyses on the PIRGs (**Supplementary Figures 2C-F**) and observed enrichment of multiple pathways, including the IL-17 and cytokine signaling pathways.

We applied the LASSO regression model to screen the key PIRGs, which resulted in the identification of 14 key PIRGs (**Figure 2D**; **Supplementary Figure 2G**). We then constructed a protein–protein interaction (PPI) network for these 14 key PIRGs, and after removing isolated protein nodes, we identified a set of hub PIRGs—LYZ, MMP9, S100A9, WNT5A, ESR1, FOS, BACH2, AQP9, and BTC—that interacted with each other (**Figure 2E**). The expression and correlation of these genes were assessed in the transcriptome (**Figures 2F, G**). To validate the accuracy of these findings, we used two independent datasets, GSE30999 and GSE78097, which confirmed that the expression levels of these 9 hub PIRGs were significantly different between psoriasis and normal tissues (**Supplementary Figure 3A, B**). Furthermore, the construction of a reliable diagnostic nomogram model using these genes demonstrated their strong diagnostic potential (**Supplementary Figure 3C, D**). Taken together, these hub PIRGs may play pivotal roles in the pathogenesis of psoriasis.

To investigate whether hAMSCs act through these hub PIRGs, we examined the expression of 9 hub PIRGs in both the control and model groups of mice (**Figures 2H–J**; **Supplementary Figure 3E–J**). In the psoriasis mouse model, the expression of 6 hub PIRGs—LYZ, MMP9, S100A9, ESR1, FOS and BACH2—was significantly different from that in the control group, and their expression trends were consistent with those observed in the human psoriasis microarray data. Notably, the expression levels of MMP9 and S100A9 were significantly lower in the hAMSC-treated group than in the model group, whereas the expression of BACH2 was significantly greater in the treatment group than in the model group. Numerous studies have demonstrated that MMP9 and S100A9 are key biomarkers associated with psoriasis. Based on these findings, we hypothesize that hAMSCs may exert therapeutic effects through modulation of MMP9, S100A9, or BACH2 positive cell in psoriatic lesions.

### 2.3 Analysis of S100A9, MMP9 and BACH2 expression in psoriatic tissues by single-cell RNA-seq

**Figure 3:**
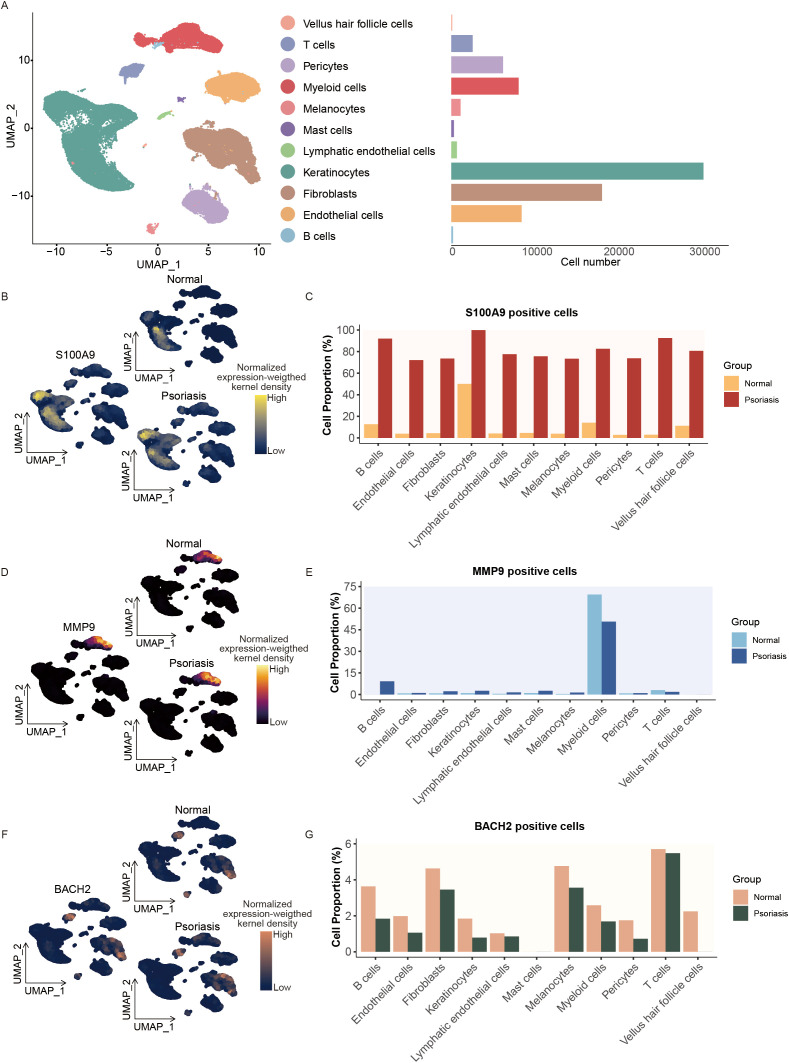
Identification of cell populations and gene signatures in psoriatic single cells. **(A)** Single-cell count matrices were obtained from the GEO database (GSE228421). The UMAP plot displays all cells, colored by their cell type identity. Bar plot showing the cell numbers of different cell types in psoriatic tissues. **(B)** UMAP plots showing the expression distribution of S100A9 in normal and psoriatic skin based on single-cell RNA sequencing data. Yellow indicates high expression and blue indicates low expression. **(C)** Proportion of S100A9 positive cells across different cell types. **(D)** UMAP plots showing the expression distribution of MMP9 in normal and psoriatic skin based on single-cell RNA sequencing data. Bright yellow indicates high expression and black indicates low expression. **(E)** Proportion of MMP9 positive cells across different cell types. **(F)** UMAP plots showing the expression distribution of BACH2 in normal and psoriatic skin based on single-cell RNA sequencing data. Orange indicates high expression and blue indicates low expression. **(G)** Proportion of BACH2 positive cells across different cell types.

Due to multiple safety and ethical constraints, it is challenging to directly employ hAMSCs in psoriatic patients to investigate their *in vivo* mechanisms. Therefore, we utilized single-cell RNA sequencing (scRNA-seq) data from psoriatic skin tissues to analyze changes in MMP9, S100A9, or BACH2 positive cells that might be affected by hAMSCs, aiming to predict the potential impact of hAMSCs in humans and their roles across different cell types. Based on the clinical information of patients, we selected 10 single-cell transcriptome datasets derived from five psoriatic skin lesions and their corresponding normal skin areas. After rigorous quality control and the exclusion of low-quality or doublet cells, a total of 88,657 cells were retained for downstream analysis. At a clustering resolution of 0.4, the cells were divided into 22 distinct clusters, each exhibiting considerable transcriptional heterogeneity. According to canonical marker genes reported in previous studies, we annotated 11 major cell types: Keratinocytes (KRT1, KRT14, KRT10, KRT5), Fibroblasts (DCN, LUM, COL1A2), Myeloid cells (LYZ, CD68, CD14), Pericytes (ACTA2, RGS5, PDGFRB, MCAM), Endothelial cells (KDR, VWF, SELE, CD93), Lymphatic endothelial cells (LYVE1, CCL21), Vellus hair follicle cells (SOX9, KRT6B, SFRP1), T cells (CD3D, CD3E, CD2), Mast cells (TPSB2, TPSAB1, CPA3, IL1RL1), Melanocytes (TYRP1, PMEL, MLANA), and B cells (CD79A, MS4A1) (**Figure 3A**; **Supplementary Figures 4A, B**).

To elucidate the transcriptional differences between psoriatic and normal tissues, we identified DEGs across cell clusters. The results revealed significant transcriptional alterations in both immune and non-immune cells of psoriatic tissues compared with normal controls (|logFC| > 0.25, adjusted P < 0.05) (**Supplementary Figure 4C**). Among all cell types, Keratinocytes, Pericytes, Fibroblasts, and Myeloid cells were most affected during psoriasis progression (**Supplementary Figure 4D**). GO enrichment analysis of upregulated DEGs showed strong enrichment in epidermal differentiation-related processes such as epidermis development, keratinocyte differentiation, epidermal cell differentiation, and cornified envelope formation (**Supplementary Figure 4E**), suggesting that multiple cell types contribute to altered keratinocyte differentiation and cornification. Conversely, downregulated genes were enriched in pathways related to cytosolic ribosome, cytoplasmic translation, and regulation of CD4-positive, alpha-beta T cell activation (**Supplementary Figure 4E**), indicating marked alterations in metabolism, translation, and immune regulation in psoriatic cells.

To further explore the cell type–specific expression patterns of S100A9, MMP9, and BACH2 in psoriasis, we systematically examined their single-cell expression profiles and compared the distribution of positive cells between psoriatic and normal tissues. The results demonstrated that S100A9 expression was predominantly enriched in keratinocytes (**Figure 3B**), and S100A9 positive cells were markedly increased in multiple cell types of psoriatic tissues, including Keratinocytes, Fibroblasts, T cells, and Myeloid cells (**Figure 3C**). Notably, nearly all keratinocytes from psoriatic lesions were S100A9 positive, suggesting a pronounced inflammatory activation state in these cells. MMP9 expression was mainly concentrated in myeloid cells (**Figure 3D**); however, compared with normal tissues, the proportion of MMP9 positive cells was reduced in psoriatic lesions (**Figure 3E**), while their overall expression level within myeloid cells was increased (**Supplementary Figure 4F**), implying that MMP9 may be highly upregulated in specific subclusters despite a lower overall frequency. In contrast, BACH2 expression was primarily localized to myeloid cells and T cells (**Figure 3F**), and the proportions of BACH2 positive cells within both populations were lower in psoriatic tissues than in normal controls (**Figure 3G**). We observed largely consistent results in the validation dataset compared with the discovery cohort. However, we unexpectedly noted an increased proportion of MMP9-positive cells within the myeloid compartment of psoriasis samples in the validation dataset (**Supplementary Figure 4F**). We speculate that this discrepancy may be attributable to differences in cell capture efficiency during the single-cell sequencing process, as the number of captured cells from psoriasis samples was relatively limited in the validation cohort, which could introduce proportional bias in specific cell subsets. Collectively, these results suggest that S100A9, MMP9, and BACH2 may play crucial roles in psoriasis pathogenesis by influencing the functions of keratinocytes, fibroblasts, T cells, and myeloid cells, providing important insights into their potential pathophysiological significance.

### 2.4 Correlation between CD8-IL17A T cells and S100A9 expression

**Figure 4:**
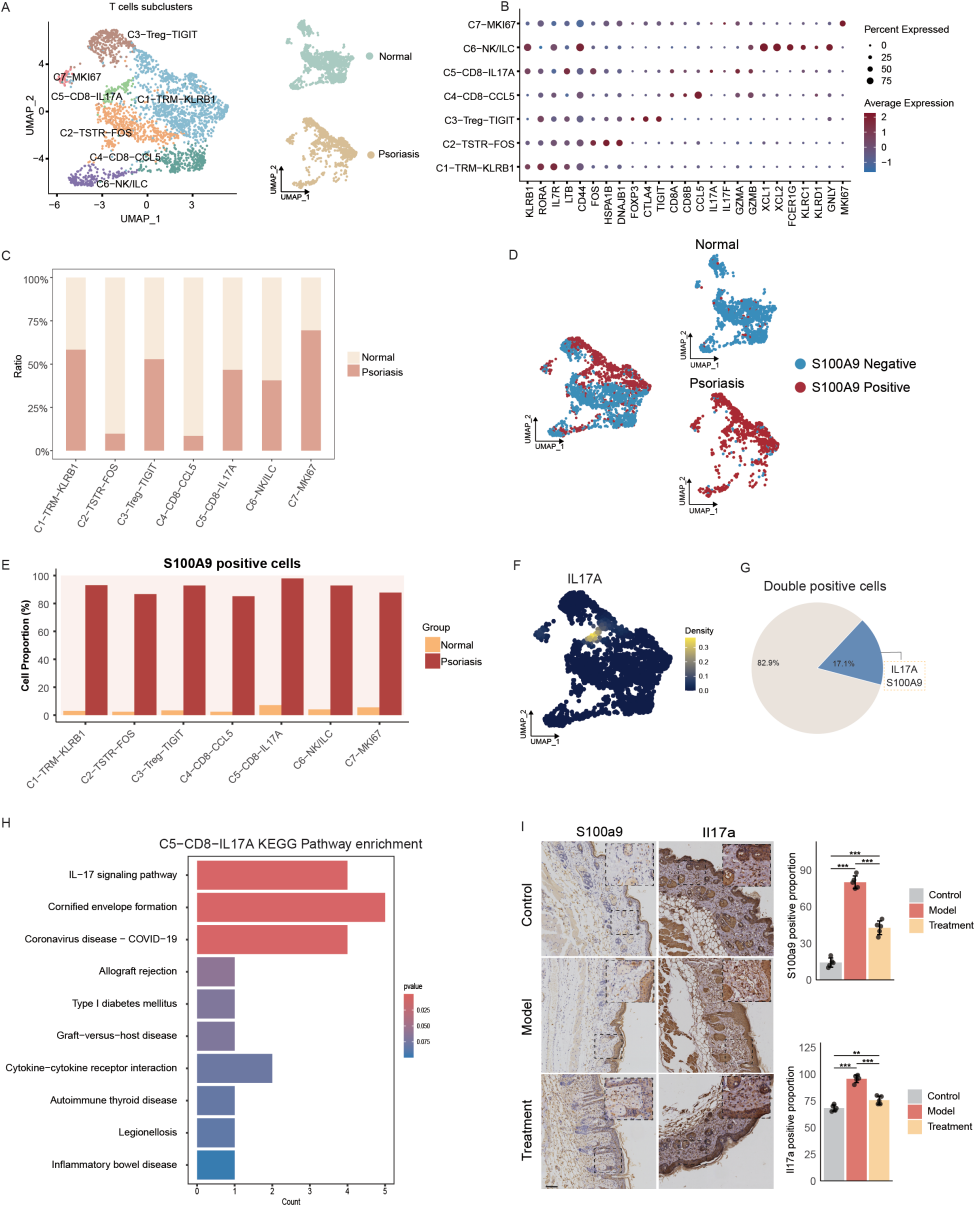
Functional annotation of T cell subclusters. **(A)** UMAP visualization of T cell subclusters (left) and their distribution in normal versus psoriatic tissues (right); TRM (Tissue-resident memory T cells), TSTR (T cell stress response state), Treg (Regulatory T cells), NK/ILC (Natural Killer cells/Innate lymphoid cells). **(B)** Bubble diagram showing characteristic genes in T cell subclusters. **(C)** Bar plot showing the cell numbers of T cell subclusters. **(D)** UMAP plots showed the total distribution of MMP9 positive T cells (left) and their distribution in normal versus psoriasis (right). **(E)** Bar plots showing the proportions of and S100A9 positive cells across different T cells types. **(F)** UMAP plots showing the expression distribution of IL17A in psoriatic skin based on single-cell RNA sequencing data. Yellow indicates high expression and blue indicates low expression. **(G)** Pie chart shows the percentage of double positive cells expressing S100A9 and IL17A in T cells of psoriasis. **(H)** Bar plot showing the enrichment of KEGG terms or pathways of C5-CD8-IL17A. **(I)** S100a9 and Il17a expression detected by IHC (n=5) (Scale bars, 100μm).

To further investigate the distribution characteristics and potential functions of S100A9, BACH2, and MMP9 positive cells within T cells, we performed subclustering of the T cell population and identified seven distinct subclusters: C1–TRM–KLRB1, C2–TSTR–FOS, C3–Treg–TIGIT, C4–CD8–CCL5, C5–CD8–IL17A, C6–NK/ILC, and C7–MKI67 (**Figures 4A, B**). The proportions of C2–TSTR–FOS and C4–CD8–CCL5 subclusters were markedly decreased in psoriatic tissues compared with normal controls (**Figure 4C**). Differential gene expression analysis revealed that C1–TRM–KLRB1 and C3–Treg–TIGIT subclusters exhibited the greatest transcriptional differences between psoriatic and normal T cells (**Supplementary Figure 5A**). KEGG pathway enrichment further demonstrated that the DEGs from both subclusters were significantly enriched in the IL-17 signaling pathway (**Supplementary Figures 5B, C**). Moreover, compared with normal controls, S100A9 expression was consistently upregulated across all T-cell subclusters in psoriatic tissues (**Supplementary Figure 5D**), suggesting that S100A9 may serve as an important marker of psoriatic T cells.

We found that S100A9 positive cells were predominantly distributed in the C1–TRM–KLRB1, C3–Treg–TIGIT, and C5–CD8–IL17A subclusters (**Figure 4D**), and these cells were more abundant in psoriatic T cells than in normal controls (**Figure 4E**). In contrast, MMP9 and BACH2 positive cells were less frequently detected across T cell subclusters (**Supplementary Figures 5E, F**), indicating that S100A9 may play a more prominent role in T cell biology during psoriasis. Notably, within the C5–CD8–IL17A cluster, IL17A expression was markedly elevated only in psoriatic cells (**Figure 4F**), and this was accompanied by strong co-expression of S100A9 (**Figure 4G**). Furthermore, differential expression analysis between psoriatic and normal C5–CD8–IL17A cells revealed that DEGs were significantly enriched in the IL-17 signaling pathway (**Figure 4H**). Collectively, these results indicate a close association between S100A9 positive T cells and IL-17 signaling in psoriasis. Importantly, further experiments showed that stem cell therapy markedly reduced the protein levels of S100A9 and IL17A in a psoriatic mouse model (**Figure 4I**), suggesting that stem cells may exert therapeutic effects in psoriasis partly by reducing the abundance of IL17A-high T cells.

### 2.5 MMP9 expression in myeloid cells

**Figure 5:**
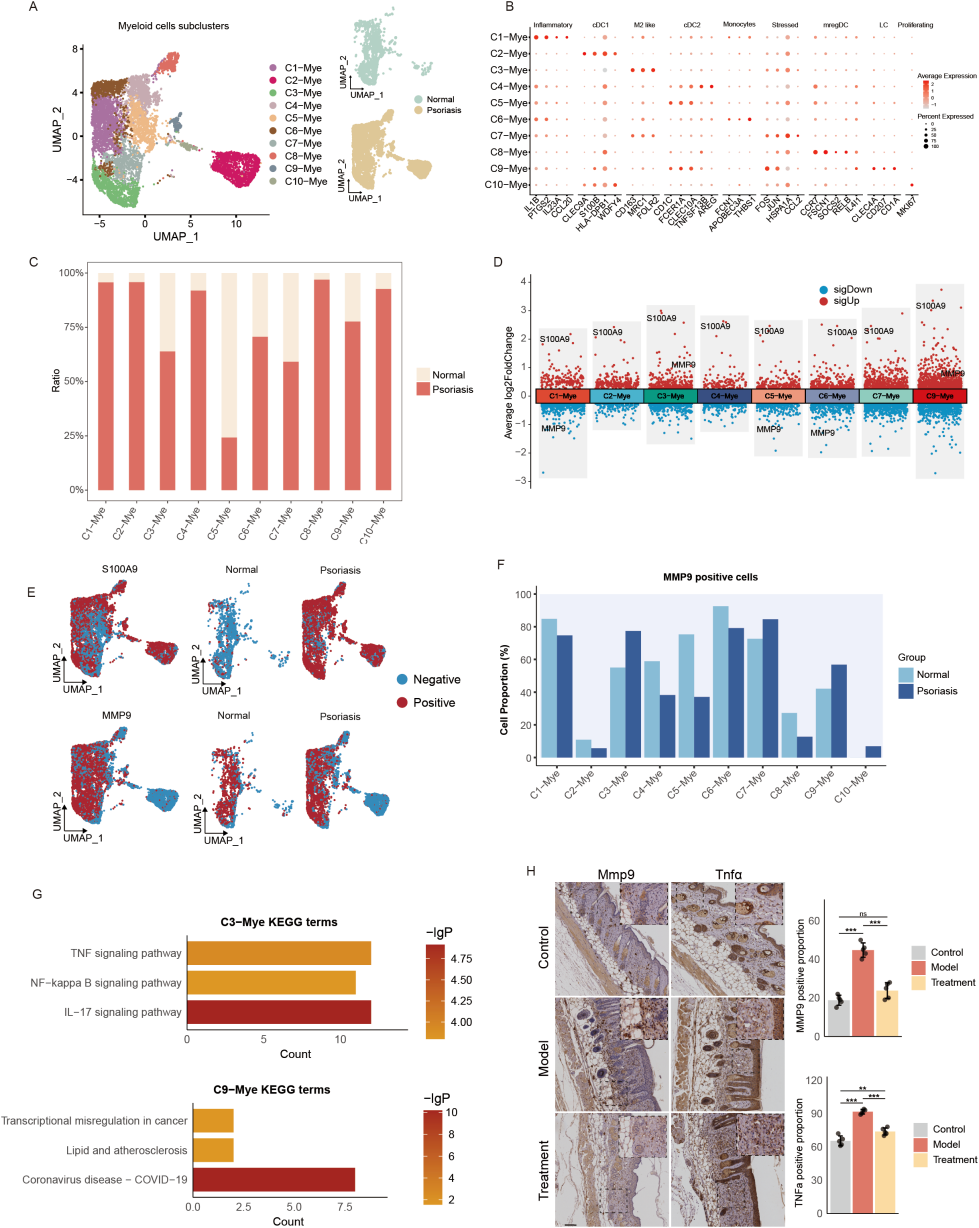
Functional annotation of myeloid cell subclusters. **(A)** UMAP visualization of myeloid cell subclusters (left) and their distribution in normal versus psoriatic tissues (right). **(B)** Bubble diagram showing characteristic genes in myeloid cell subclusters. **(C)** Bar plot showing the cell numbers of myeloid cell subclusters. **(D)** The dot plot illustrates the differential gene expression across myeloid cell subclusters. **(E)** UMAP plots showed the total distribution of MMP9 positive myeloid cells (left) and their distribution in normal versus psoriasis (right). **(F)** Bar plots showing the proportions of and MMP9 positive cells across different myeloid cells types. **(G)** Bar plot showing the enrichment of KEGG terms or pathways of C3-Mye and C9-Mye with DEGs of MMP9 positive and MMP9 negative cells. **(H)** Mmp9 and Tnfα expression detected by IHC (n=5) (Scale bars, 100μm).

We next analyzed the distribution and potential functions of S100A9, BACH2, and MMP9 positive cells within the myeloid compartment. A total of ten myeloid subclusters were identified: C1–Mye, C2–Mye, C3–Mye, C4–Mye, C5–Mye, C6–Mye, C7–Mye, C8–Mye, C9–Mye, and C10–Mye (**Figure 5A**; **Supplementary Figure 6A**). Among them, C1–Mye showed high expression of inflammation and chemokine related genes (IL1B, PTGS2, IL23A, CCL20) and monocyte markers (FCN1, APOBEC3A, THBS1), suggesting an inflammatory monocyte identity. C2–Mye expressed canonical cDC1 markers (CLEC9A), while C3–Mye was enriched for M2-like macrophage markers (CD163). Both C4–Mye and C5–Mye expressed cDC2 markers (CD1C), although C4–Mye additionally showed high expression of inflammation-related genes (TNFSF13B, AREG). C6–Mye expressed monocyte markers at higher levels but with weaker inflammatory signatures than C1–Mye. C7–Mye expressed stress-response genes (FOS, JUN), C8–Mye was characterized by mature dendritic cell activation markers (CCR7, FSCN1), C9–Mye displayed Langerhans cell markers (CLEC4A, CD1A), and C10–Mye expressed proliferative cDC1-related genes (MKI67, CLEC9A) (**Figure 5B**). In psoriatic tissues, C1–Mye, C2–Mye, C4–Mye, C8–Mye, C9–Mye, and C10–Mye were significantly enriched, whereas C5–Mye was notably reduced (**Figure 5C**).

Compared with normal skin, psoriatic samples showed widespread upregulation of S100A9 across myeloid subclusters; C3–Mye and C9–Mye displayed prominent upregulation of MMP9, while C1–Mye, C5–Mye, and C6–Mye showed MMP9 downregulation, and BACH2 expression remained largely unchanged (**Figure 5F**). S100A9 positive cells were predominantly localized in psoriatic lesions but were rarely detected in normal skin (**Figure 5D**; **Supplementary Figure 6C**). KEGG enrichment analysis revealed that DEGs from S100A9 positive cells were significantly enriched in the IL-17 signaling pathway (**Supplementary Figure 6D**). MMP9 positive cells were detected in both normal and psoriatic tissues; however, their proportions increased notably in C3–Mye, C7–Mye, C9–Mye, and C10–Mye, while decreasing in other subclusters (**Figure 5D, E**). Together with our previous findings showing elevated MMP9 expression in psoriatic patients and mouse models, these results suggest that C3–Mye and C9–Mye may play key roles in psoriasis pathogenesis. Differential gene expression analysis between MMP9 positive and negative cells showed that DEGs in C3–Mye were enriched in the IL-17 signaling, TNF signaling, and NF-κB signaling pathways, whereas DEGs in C9–Mye were enriched in Coronavirus disease–COVID-19, Transcriptional misregulation in cancer, and Lipid and atherosclerosis pathways (**Figure 5G**), indicating a core role of C3–Mye in inflammatory and immune regulation in psoriasis. BACH2 positive cells were scarce and observed in small numbers in both normal and psoriatic tissues (**Supplementary Figure 6B**). Importantly, immunohistochemistry confirmed that stem cell therapy markedly reduced MMP9 and TNFα protein levels in psoriatic mouse models (**Figure 5H**), suggesting that stem cells may exert therapeutic effects by modulating the TNF signaling pathway.

### 2.6 S100A9 positive fibroblasts and their association with inflammatory pathways

**Figure 6:**
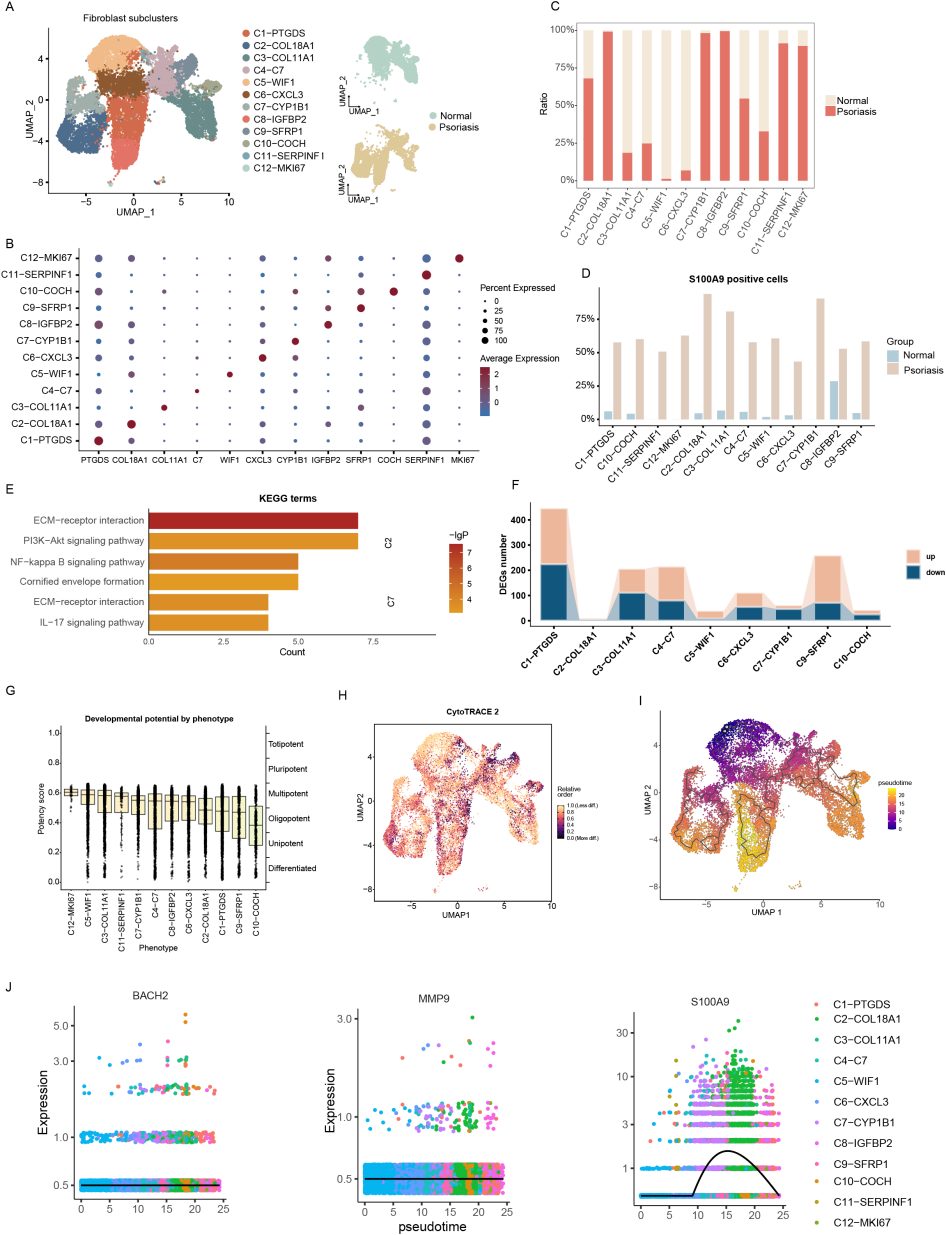
Functional annotation of fibroblast cell subclusters. **(A)** UMAP visualization of fibroblast subclusters (left) and their distribution in normal versus psoriatic tissues (right). **(B)** Bubble diagram showing characteristic genes in fibroblast subclusters. **(C)** Bar plot showing the cell numbers of fibroblast subclusters. **(D)** Bar plots showing the proportions of and S100A9 positive cells across different fibroblast subclusters. **(E)** Bar plot showing the enrichment of KEGG terms or pathways of C2-COL18A1 and C7-CYP1B1 with feature genes. **(F)** Bar plot showing the numbers of DEGs across different fibroblast subclusters. **(G)** Boxplots showing CytoTRACE research of the developmental potential of fibroblast subclusters. **(H)** CytoTRACE analysis among fibroblast subclusters. **(I)** UMAP plot showing the distribution of differentiation trajectories of fibroblasts in fibroblast subclusters. **(J)** Expression dynamics of BACH2, MMP9, and S100A9 along the pseudotime trajectory.

We next examined the distribution and potential functions of S100A9, BACH2, and MMP9 positive cells among non-immune cell populations. Fibroblasts were further subdivided into 12 transcriptionally distinct subclusters: C1–PTGDS, C2–COL18A1, C3–COL11A1, C4–C7, C5–WIF1, C6–CXCL3, C7–CYP1B1, C8–IGFBP2, C9–SFRP1, C10–COCH, C11–SERPINF1, and C12–MKI67 (**Figure 6A, B**). In psoriatic tissues, C1–PTGDS, C2–COL18A1, C7–CYP1B1, C8–IGFBP2, C11–SERPINF1, and C12–MKI67 were significantly enriched, whereas C3–COL11A1, C4–C7, C5–WIF1, C6–CXCL3, and C10–COCH were reduced (**Figure 6C**). S100A9 positive fibroblasts were mainly distributed in C2–COL18A1 and C7–CYP1B1 subclusters, both of which were enriched in psoriatic tissues (**Figure 6D**; **Supplementary Figure 7A**). MMP9 positive cells were also concentrated in these two subclusters (**Supplementary Figure 7B**), whereas BACH2 positive cells were primarily localized to C3–COL11A1 and were less abundant overall (**Supplementary Figure 7C**).

Functional enrichment analysis showed that C2–COL18A1 genes were significantly enriched in ECM–receptor interaction, PI3K–Akt signaling, and NF-κB signaling pathways, while C7–CYP1B1 genes were associated with cornified envelope formation, ECM–receptor interaction, and IL-17 signaling (**Figure 6E**). Comparison between psoriatic and normal fibroblasts revealed that C1–PTGDS and C9–SFRP1 had the largest number of DEGs (**Figure 6F**; **Supplementary Figure 7D**). GO enrichment of upregulated genes highlighted extracellular matrix remodeling and immune activation, including collagen-containing extracellular matrix, humoral immune response, and myeloid leukocyte migration, suggesting an activated state associated with inflammation and tissue repair. Downregulated genes were enriched in cytosolic ribosome and translation-related processes, indicating decreased biosynthetic activity (**Supplementary Figure 7E**).

Developmental trajectory analysis using CytoTRACE and pseudotime ordering revealed clear differentiation hierarchies among fibroblast subtypes. C12–MKI67 showed the highest developmental potential, suggesting a proliferative or progenitor-like state; C5–WIF1, C3–COL11A1, and C11–SERPINF1 represented intermediate stages, while C10–COCH and C9–SFRP1 displayed the lowest potential, representing terminally differentiated states (**Figure 6G, H**). Pseudotime analysis revealed a continuous differentiation spectrum, transitioning from early C5–WIF1 states to terminal C2–COL18A1, C8–IGFBP2, and C10–COCH states (**Figure 6I**). Dynamic gene expression analysis indicated stable expression of BACH2 and MMP9 throughout differentiation, whereas S100A9 expression markedly increased during late stages, suggesting its critical role in inflammatory fibroblast activation (**Figure 6J**). Overall, S100A9 positive fibroblasts were primarily enriched in C2–COL18A1 and C7–CYP1B1—two psoriasis-specific clusters strongly associated with inflammatory and immune pathways—implying a central regulatory role of S100A9 in fibroblast-mediated inflammatory remodeling in psoriasis.

### 2.7 Keratinocyte subclusters and their relationship with NF-κB related inflammation

**Figure 7:**
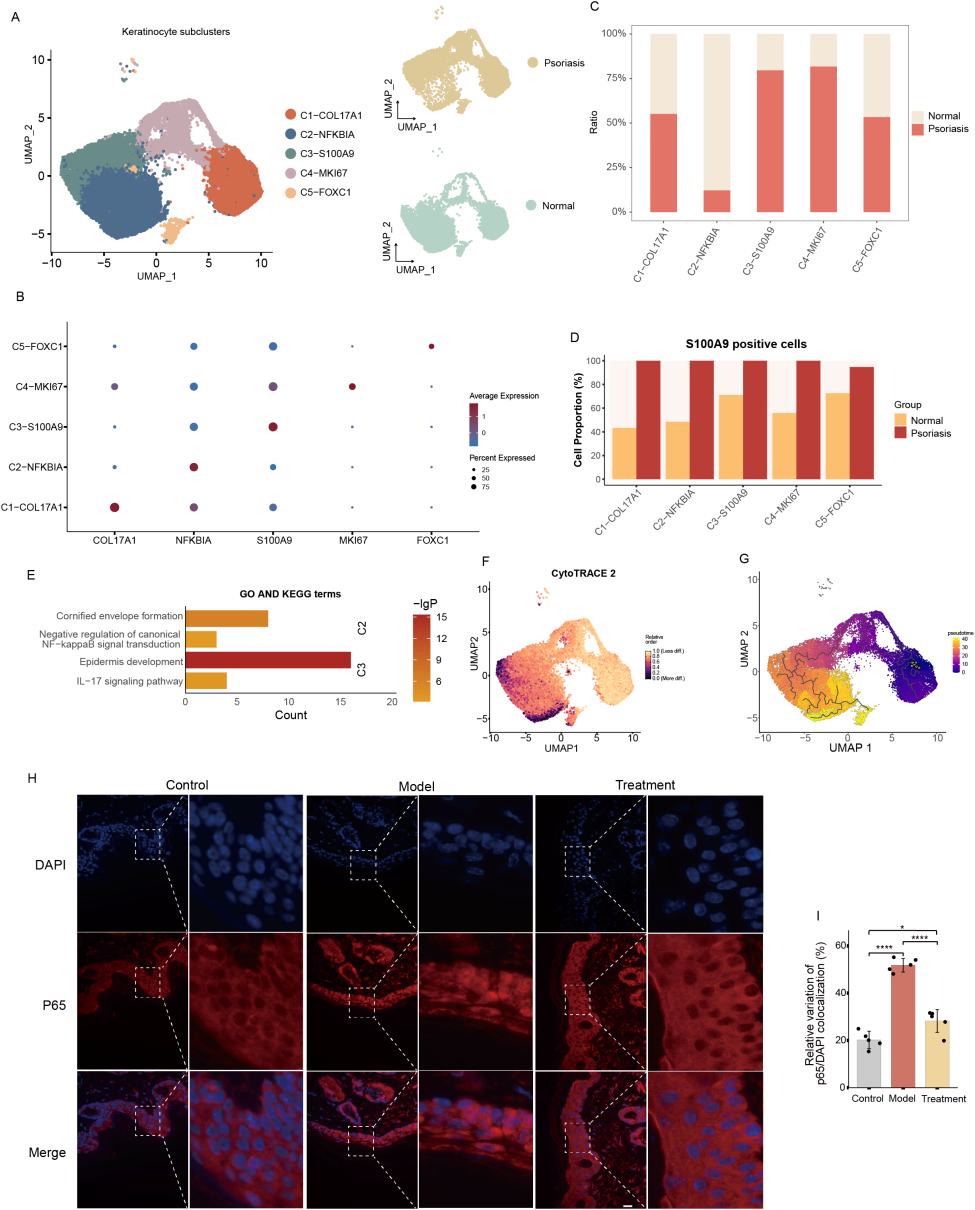
Functional annotation of keratinocyte cell subclusters. **(A)** UMAP visualization of keratinocyte subclusters (left) and their distribution in normal versus psoriatic tissues (right). **(B)** Bubble diagram showing characteristic genes in keratinocyte subclusters. **(C)** Bar plot showing the cell numbers of keratinocyte subclusters. **(D)** Bar plots showing the proportions of and S100A9 positive cells across different keratinocyte subclusters. **(E)** Bar plot showing the enrichment of KEGG terms or pathways of C2-NFKBIA and C3-S100A9 with feature genes. **(F)** CytoTRACE analysis among keratinocyte subclusters. **(G)** UMAP plot showing the distribution of differentiation trajectories of fibroblasts in keratinocyte subclusters. **(H, I)** Immunofluorescence staining for p65 was performed on the tissues of the control group, model group and treatment group respectively, and the nuclear translocation of p65 was quantitatively analyzed. (n=5) (Scale bars, 20μm). Each experiment was repeated at least three times. ns not significant, *P< 0.05, ****P < 0.0001.

Keratinocytes were further subdivided into five subclusters: C1–COL17A1, C2–NFKBIA, C3–S100A9, C4–MKI67, and C5–FOXC1 (**Figure 7A, B**). Compared with normal skin, C3–S100A9 and C4–MKI67 were markedly increased in psoriatic tissues, whereas C2–NFKBIA was significantly reduced (**Figure 7C**). Although MMP9- and BACH2 positive cells were relatively rare among keratinocytes, their distributions were distinct: MMP9 positive cells were mainly located in C1–COL17A1, C3–S100A9, and C4–MKI67, while BACH2 positive cells were enriched in the C2–NFKBIA subcluster of normal skin (**Supplementary Figure 8B, C**). S100A9 positive cells were widely distributed across keratinocyte subclusters—particularly C3–S100A9 and C4–MKI67—and to a lesser extent in C2–NFKBIA (**Figure 7D**; **Supplementary Figure 8A**). The NFKBIA gene encodes IκBα, a negative regulator of the NF-κB signaling pathway, which limits excessive inflammatory activation via negative feedback. We therefore hypothesized that the C2–NFKBIA cluster may play an anti-inflammatory regulatory role in psoriasis. Differential gene enrichment analysis supported this notion, showing that C2–NFKBIA DEGs were enriched in cornified envelope formation and negative regulation of canonical NF-κB signaling, while C3–S100A9 DEGs were enriched in epidermis development and the IL-17 signaling pathway (**Figure 7E**). These results highlight strong associations between C2–NFKBIA and C3–S100A9 with inflammation and immune responses.

Trajectory analysis revealed that C1–COL17A1 and C4–MKI67 had the highest developmental potential, indicating undifferentiated or proliferative states, while C5–FOXC1, C3–S100A9, and C2–NFKBIA represented more differentiated stages (**Figure 7F**; **Supplementary Figure 8D**). Pseudotime ordering showed that keratinocyte differentiation progressed from early C5–FOXC1 and C4–MKI67 stages toward terminal C2–NFKBIA states (**Figure 7G**). Dynamic gene expression analysis revealed stable BACH2 and MMP9 expression but a marked upregulation of S100A9 in late stages, suggesting its role in inflammatory keratinocyte activation.

Taken together, these results indicate that both fibroblasts and keratinocytes exhibit strong associations between S100A9 positive cell states and NF-κB–related signaling. We propose that hAMSCs may ameliorate psoriasis by modulating NF-κB activity. Consistently, immunofluorescence analysis of psoriatic mouse models showed that stem cell therapy markedly reduced nuclear translocation of p65 (**Figure 7H, I**), confirming that hAMSCs effectively suppress NF-κB pathway activation in psoriasis.

## 3 Discussion

In recent years, extensive research has been conducted on MSCs in the field of dermatology, with notable achievements, particularly in skin immune-related diseases (11, 22). Owing to their unique immunomodulatory properties and tissue regenerative capacity (23), MSCs have demonstrated therapeutic potential in various dermatological conditions, including atopic dermatitis (24, 25). Moreover, MSC-based therapies have shown favorable outcomes in skin rejuvenation, hair loss treatment, and scar reduction (26, 27). A growing body of evidence has also reported the significant efficacy of stem cell–based therapies in psoriasis, although the underlying mechanisms of action and long-term safety profiles remain incompletely understood (22). In the present study, we established a mouse model of psoriasis-like skin lesions to systematically evaluate the therapeutic efficacy and safety of human amniotic mesenchymal stem cells (hAMSCs) in psoriasis. By integrating GEO datasets with ImmPort resources and applying a LASSO-based feature selection strategy, we comprehensively identified immune-related hub genes associated with psoriasis. Furthermore, single-cell RNA sequencing was employed to generate a high-resolution cellular atlas of S100A9-, MMP9-, and BACH2-positive populations within the immune and stromal compartments. Our results demonstrate that the therapeutic effects of hAMSCs are predominantly mediated through modulation of the S100A9–IL-17–TNF–NF-κB signaling axis, leading to a marked reduction in IL17A-high CD8^+^ T cells, inflammatory myeloid subsets, and S100A9-high fibroblast/keratinocyte states. Collectively, these findings provide a strong experimental and mechanistic foundation for the development of hAMSC-based therapeutic strategies for psoriasis.

The treatment of psoriasis encompasses multiple therapeutic approaches, primarily including topical therapy, phototherapy, oral systemic therapies, and biologics (1). Compared with traditional therapies, biologics achieve targeted inhibition of key inflammatory factors (e.g., IL-17 and TNF-α), not only significantly improving skin lesion clearance rates but also demonstrating favorable safety profiles (28, 29). These groundbreaking advances have enabled long-term disease management for moderate-to-severe patients, fundamentally transforming the therapeutic landscape of psoriasis. However, limitations remain, including suboptimal responses in some patients and high recurrence rates after discontinuation (30). In contrast to currently approved biologic agents that primarily target single inflammatory pathways, such as TNF-α, IL-17, or IL-23, human amniotic mesenchymal stem cells (hAMSCs) exert broader immunomodulatory effects. hAMSCs can secrete multiple immunoregulatory factors, suppress pro-inflammatory cytokine production, promote the expansion of regulatory T cells (Tregs), and remodel the immune microenvironment, thereby attenuating inflammatory responses in a more holistic manner (15). In addition, hAMSCs may facilitate tissue repair and the restoration of immune homeostasis. However, existing studies on hAMSCs have largely been confined to imiquimod (IMQ)-induced psoriasis-like animal models, and robust clinical evidence supporting their translational application remains limited (31). In this study, we evaluated hAMSC treatment in a psoriasis mouse model. The administration of hAMSCs on day 0 significantly delayed the onset of psoriatic lesions, whereas treatment initiated on day 4 markedly alleviated lesion severity in the intervention group, suggesting that hAMSCs are effective in both the prevention and treatment of psoriasis in the model mice (**Figure 1C**). hAMSCs demonstrated a favorable safety profile in psoriasis therapy, causing no significant impairment in the liver, which is consistent with findings by Wen et al. (32), Imai et al. (19), and Shi et al. (12). Furthermore, emerging evidence indicates that MSC-derived exosomes also exhibit therapeutic benefits in psoriasis management (25). These findings collectively underscore the therapeutic potential of both MSCs and MSC-derived exosomes in psoriasis, laying the foundation for the development of MSC-based therapeutics for this condition.

In this study, we screened immune-related genes associated with psoriasis and found that some of these genes play significant roles in regulating cytokine-related signaling pathways in psoriasis (33). Experimental validation revealed that the expression levels of MMP9, S100A9, and BACH2 were significantly altered following treatment with hAMSCs. Based on these findings, we further analyzed MMP9-, S100A9-, and BACH2-positive cells in single-cell transcriptomic data from human psoriatic skin to predict the potential effects of hAMSCs in humans and their functions in different cell types. BACH2 is a transcription factor that serves as a central regulator of immune cell differentiation and function, particularly in T and B lymphocytes (34). In our analysis, BACH2-positive cells were mainly distributed in T cells and myeloid cells; however, their overall abundance in psoriatic lesions was low, and thus, we did not perform further analyses. Previous studies by Liu et al. (35) demonstrated through ELISA that BACH2 expression levels were significantly reduced in psoriasis patients compared to healthy controls, suggesting that BACH2 may play an important immunoregulatory role in psoriasis.

Previous studies on MMP9 have primarily focused on neutrophils, in which MMP9 is released to disrupt tight junctions and cytoskeletal integrity, thereby inducing vascular barrier dysfunction and increased permeability (36). However, we did not identify a neutrophil cluster in our psoriasis single-cell dataset, likely due to the fragility of neutrophils, which are difficult to capture in early single-cell sequencing experiments (37). Our data showed that MMP9 was predominantly expressed in myeloid cells, but its upregulation was not uniform across all myeloid subtypes. Notably, MMP9 expression was markedly elevated in the C3-Mye cluster, which also exhibited high expression of M2 macrophage-associated markers, suggesting that MMP9 may also play a crucial role in M2-like macrophages in psoriasis. Moreover, C3-Mye cells were enriched in multiple inflammatory signaling pathways, further supporting the key involvement of M2-like macrophages in the pathogenesis of psoriasis.

Under inflammatory conditions, the calcium-binding proteins S100A8 and S100A9 are typically upregulated, forming either homodimers or heterodimers (S100A8/A9), collectively known as calprotectin (CP) (38, 39). During cutaneous inflammation, S100A9 is mainly expressed by keratinocytes, neutrophils, and macrophages (40). Our single-cell analysis of human psoriatic skin revealed that S100A9 expression was elevated in multiple cell types, particularly in keratinocytes, fibroblasts, T cells, and myeloid cells. Furthermore, S100A9-positive T cells, myeloid cells, fibroblasts, and keratinocytes were all significantly enriched in the IL17A signaling pathway, suggesting a strong link between S100A9 expression and IL17A-mediated inflammation. In addition, S100A9-positive fibroblasts and keratinocytes were enriched in NF-κB–related signaling pathways, indicating that S100A9 is involved in multiple inflammatory pathways in psoriasis.

In summary, this study demonstrates the therapeutic efficacy and safety of hAMSCs in the treatment of psoriasis. Through comprehensive bioinformatic analyses, we further characterized the dynamic alterations of MMP9-, S100A9-, and BACH2-positive cell populations, which may underlie the therapeutic effects of hAMSCs. Notably, we propose MMP9, S100A9, and BACH2 as potential biomarkers for monitoring responses to stem cell–based therapy and for predicting the functional roles of hAMSCs *in vivo*. These findings provide novel mechanistic insights and a theoretical foundation for the clinical application of stem cell therapy. However, this study has several limitations. First, due to ethical and safety concerns, the experimental validation was conducted primarily in mouse models, which limits the direct extrapolation of our findings to humans. Second, the specific bioactive components responsible for the therapeutic effects of hAMSCs remain unidentified, representing an important direction for future research.

## 4 Conclusions

In conclusion, our findings demonstrate the therapeutic potential and underlying mechanisms of hAMSCs in psoriasis management, suggesting new therapeutic alternatives for this condition.

## 5 Materials and methods

### 5.1 Culture of hAMSCs

hAMSCs were provided by Jilin Zhongke Bio-engineering Joint Stock Co., Ltd. (Jilin, China), and cultivated in Showninm -ncMissionBasal Medium V3.0 (Shownin, China) at 37°C with 5 % CO2. *In vivo* injection into a mouse model and *in vitro* co-culture experiments using passage 4 hAMSCs.

### 5.2 Psoriasis mouse models

Twenty-five female C57BL/6 mice (8–10 weeks; Vital River Experimental Animal Technical Co., Ltd., Beijing, China) were randomly divided into three groups: Control (control group, n=5), IMQ induced model (model group, n=10) and human amniotic mesenchymal stem cells (hAMSCs) treated model (treatment group, n=10). On day 0 (1 day before modelling), dorsal hairs were removed using depilatory cream. From day 1 to day 6, mice in the model group and treatment group received daily topical application of 62.5 mg 5% imiquimod cream to the depilated skin, while mice in the control group received equivalent amounts of petroleum jelly ointment. The treatment group received intravenous injection of hAMSCs (2×106 cells in 100 μL PBS) on day 0 and day 4. Daily photos were taken to observe the changes of skin lesions on the backs of mice. The mice were anesthetized with 2% isoflurane and subsequently euthanized by cervical dislocation, in strict accordance with ethical guidelines, to ensure a humane and painless death. The Animal Ethics Committee of Changchun Sci-Tech University also examined and approved all experimental procedures and techniques (Protocol No. CKARI202409). We housed all mice under standard laboratory conditions in temperature-controlled rooms and provided them with 12 h of light and 12 h of darkness every day. The animals were normally raised without any abnormal deaths. The work has been reported in line with the ARRIVE guidelines 2.0.

### 5.3 Psoriasis area severity index score evaluation

The PASI scoring system was used to assess the inflammatory status of the dorsal skin of the mice over an 8-day period. The three parameters evaluated were erythema (redness), induration (thickness), and desquamation (scaling). Each parameter was scored from 0 to 4 (0 = none, 1 = slight, 2 = moderate, 3 = marked, 4 = very marked), yielding a cumulative score ranging from 0 to 12. The evaluations were independently conducted by two researchers, and the mean scores were calculated.

### 5.4 Cell culture and TNFα stimulation

hAMSCs were obtained from Jilin Zhongke Bio-engineering Joint Stock Co.,Ltd. (Changchun, Jilin, China), and HaCaT cells were provided by the School of Life Sciences, Jilin University. HaCaT cells were cultured in 6-well plates (Corning, 3516) at 37°C with 5% CO_2_ in DMEM (Gibco, USA) supplemented with 10% fetal bovine serum (Gibco, USA) and 100 U/ml penicillin and 100 μg/ml streptomycin. The psoriatic keratinocyte model was induced by adding TNFα to the HaCaT culture medium at a final concentration of 30 ng/mL. Co-culture of hAMSCs with HaCaT cells using Labselect^®^ cell culture chambers (LABSELECT, 14211).

### 5.5 Hematoxylin and eosin staining and immunohistochemistry

Paraffin-embedded skin samples were stained with H&E, and histological analysis was performed using the Baker scoring system. The following criteria were used: Stratum corneum: Small pustules were scored as 2 points, incomplete keratinization as 1 point, and hyperkeratinization as 0.5 points. Stratum epidermis: Disappearance of the granular cell layer in the epidermis was scored as 1 point, and hypertrophy of the stratum spinosum as 1 point. Stratum dermis: Capillary dilation was scored as 5 points, and single polymorphonuclear cell infiltration was scored as 2, 1, or 0.5 points, depending on severity. Immunohistochemistry was performed following standard protocols (41). Primary antibodies used included anti-S100A9 (Servicebio, GB111079) and anti-Ki-67 (Cell Signaling Technology, #90298).

### 5.6 Enzyme-linked immunosorbent assay

Blood was collected from anesthetized mice via retro-orbital bleeding before euthanasia. Samples were centrifuged twice at 16,000 × g for 10 minutes, and the supernatant was stored at -80 °C for further analysis. Serum levels of IL-17, TNF-α, ALT, and AST were quantified using ELISA kits (Ready-SET-GO, eBioscience, San Diego, CA) according to the manufacturer’s instructions.

### 5.7 RNA extraction and real-time PCR

Tissue samples were snap-frozen and ground in liquid nitrogen. Total RNA was extracted using TRIzol (TransGen Biotech, ET101) following the manufacturer’s protocol. cDNA synthesis was performed using the TransScript^®^ One-Step gDNA Removal and cDNA Synthesis SuperMix TransScript (TransGen Biotech, AT311), with 2μg of total RNA as the template. RT-PCR was performed on a Applied Biosystems real-time PCR system using PerfectStart^®^ Green qPCR SuperMix (TransGen Biotech, AQ601). **Supplementary Table S1** shows the primers used in this study. All experiments were conducted in triplicate.

### 5.8 EdU cell proliferation assay

Cell proliferation was assessed using the EdU assay. HaCaT cells (1×10^5^) were seeded in 12-well plates and incubated for 24 hours. EdU (10μM) reagent (Beyotime, C0071S) was added to each well for 2 hours. After three washes with PBS, cells were fixed with 4% paraformaldehyde (Sangon Biotech, E672002) for 15 minutes, permeabilized with 0.3% Triton X-100 (Sangon Biotech, A110694) for 15 minutes, and then incubated with a click-reaction reagent for 30 minutes in the dark. Nuclei were counterstained with Hoechst. The results were observed under a fluorescence microscope (Nikon ECLIPSE Ti-S) using NIS-Elements F v4.0 software.

### 5.9 ROS detection

ROS production was measured using a ROS detection kit (Beyotime, S0033S). Cells were incubated with 10 μM DCFH-DA for 30 minutes at 37 °C, and ROS levels were assessed by fluorescence microscopy.

### 5.10 Psoriasis transcriptome data collection

Two GEO datasets, GSE13355 (42) and GSE14905 (43), were used as the training set for data analysis. Batch effects were eliminated using the R package limma (v3.62.1) (44). The GEO datasets GSE30999 (45) and GSE78097 (46) were used as validation sets. Immune-related gene data were obtained from the ImmPort database (21) (https://www.immport.org/Shared/) , totaling 1793 immune-related genes.

### 5.11 Differential expression analysis

Data from GSE13355 and GSE14905 were combined and processed using limma to identify differentially expressed genes (DEGs) between normal and psoriasis samples, with the following cutoff: P < 0.05 and |logFC| > 1. The intersection of these DEGs with immune-related genes was visualized in Venn diagrams.

### 5.12 Functional enrichment analysis

Gene Ontology (GO) and Kyoto Encyclopedia of Genes and Genomes (KEGG) enrichment analyses of immune-related DEGs were performed using the R package clusterProfiler (47). Bubble plots for KEGG pathways and GO terms (biological process, cellular component, and molecular function) were generated using ggplot2.

### 5.13 Core gene selection

Core genes were selected using LASSO regression (R package glmnet (48)). Protein-protein interaction (PPI) network analysis was performed using the STRING database (49) (https://string-db.org/) and visualized in Cytoscape (v3.10.0).

### 5.14 Nomogram construction and validation

A nomogram model for predicting psoriasis occurrence was developed using the R package RMS. Calibration curves were plotted to assess the model’s predictive performance.

### 5.15 scRNA-seq data processing

The single-cell transcriptome dataset GSE228421 (50) from the GEO database was analyzed using the R package Seurat (51) (v4.4.0). Data normalization, quality control, clustering, and differential gene expression analysis were performed. Quality control criteria included 500-4,000 genes detected per cell, total UMIs >1,000, and mitochondrial gene percentage <15%. Doublet cells were excluded using the DoubletFinder (v2.0.3) algorithm. Batch effects were corrected using Harmony (52) (v1.2.3). Principal component analysis (PCA) was performed, followed by clustering with resolutions ranging from 0.1 to 1.2. Thirteen clusters were identified (resolution = 0.1). The R package Libra (53) was used to construct pseudobulk and screen differentially expressed genes (|logFC|>0.25, P.Value_adj < 0.05). Fibroblasts, keratinocytes, T cells, and myeloid cell populations were subjected to a second round of clustering using the same analytical pipeline as described above. Cell clustering was performed using the FindNeighbors and FindClusters functions implemented in the Seurat package. The GEO datasets GSE230842 (54) were used as validation sets.

### 5.16 Pseudotime analysis

Cellular differentiation potential was quantified through transcriptional entropy analysis using CytoTRACE (v1.1.0), a computational framework for predicting developmental potency based on gene expression heterogeneity (55). Pseudotime trajectories were generated using the R package Monocle3 (56) (v1.3.7), with continuous changes in cell state and branching structures visualized using the plot_cell function.

### 5.17 Statistical analysis

Data were analyzed using GraphPad (v 10.0.2). Two-tailed t-tests were used to compare differences between groups. Quantitative data are presented as mean ± standard error of the mean (SEM) or standard deviation (SD), with statistical significance defined as p < 0.05.

## Data Availability

The original contributions presented in the study are included in the article/**Supplementary Material**. Further inquiries can be directed to the corresponding authors.
