# Supplementary material for: Amelioration of psoriasis-like skin lesions by human amniotic mesenchymal stem cells: insights from multiomics profiling in mice
Source: Front Immunol. 2026 Feb 17;17:1776874. doi: 10.3389/fimmu.2026.1776874 (PMC12953415; doi:10.3389/fimmu.2026.1776874)
Supplement: Supplementary file 1 [file Table1.docx]

Supplementary Material

# Supplementary Figures and Tables

## Supplementary Figures


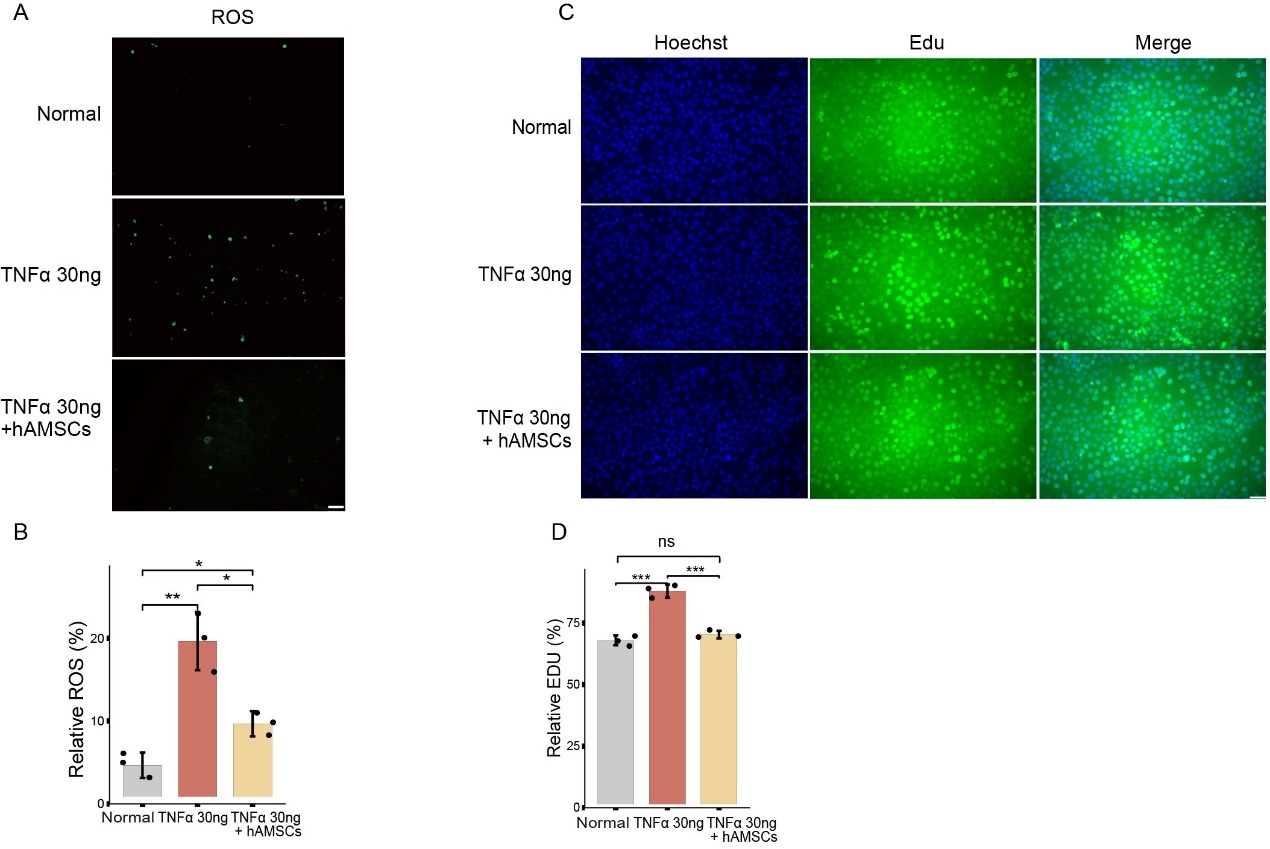


**Supplementary Figure 1.** (A-B) Fluorescence microscopy showed that hAMSCs treatment of HaCaT cells for 24 hours inhibited TNFα-induced ROS production in HaCaT cells, with quantitative analysis of ROS (n=3) (Scale bars, 100μm). (C-D) The Edu assay was used to analyze the proliferation of HaCaT, with quantitative analysis of Edu assay. Hoechst for nuclear staining (blue) (n=3) (Scale bars, 50μm). ns not significant, *P< 0.05, **P < 0.01, ***P < 0.001, ****P < 0.0001.


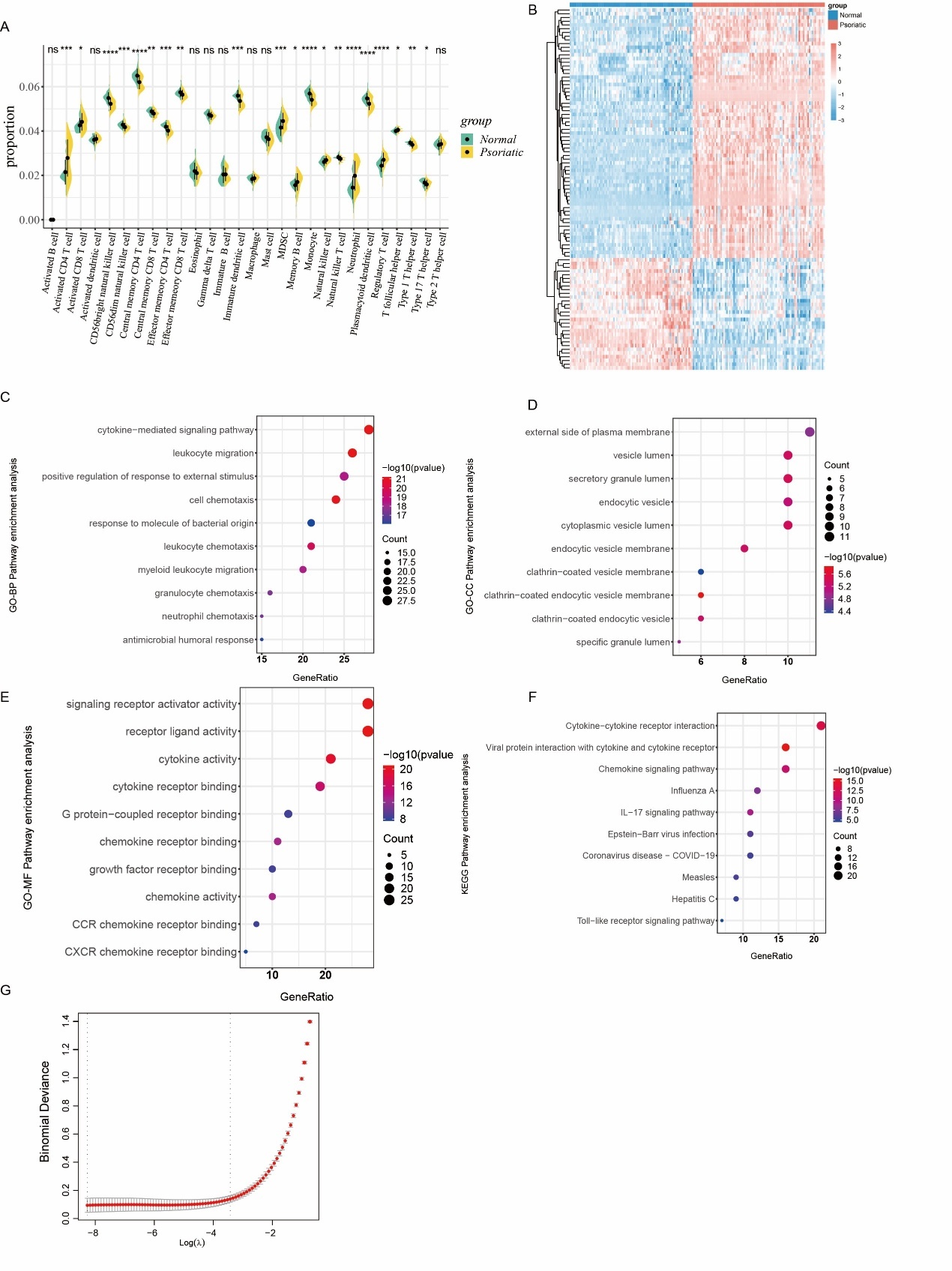


**Supplementary Figure 2.** (A) Differences in immune cell populations between normal and psoriatic tissues were assessed using the ssGSEA algorithm applied to the GSE13355 and GSE14905 datasets. (B) Heatmap of immune-related DEGs in GSE13355 and GSE14905 datasets. (C-E) GO enrichment analysis of immune-related DEGs between psoriatic and normal tissues. (F) KEGG enrichment analysis of DEGs between psoriatic and normal tissues. (G) By LASSO logistic regression algorithm, with penalty parameter tuning conducted by 10-fold cross-validation. ns not significant, *P< 0.05, **P < 0.01, ***P < 0.001, ****P < 0.0001.

**
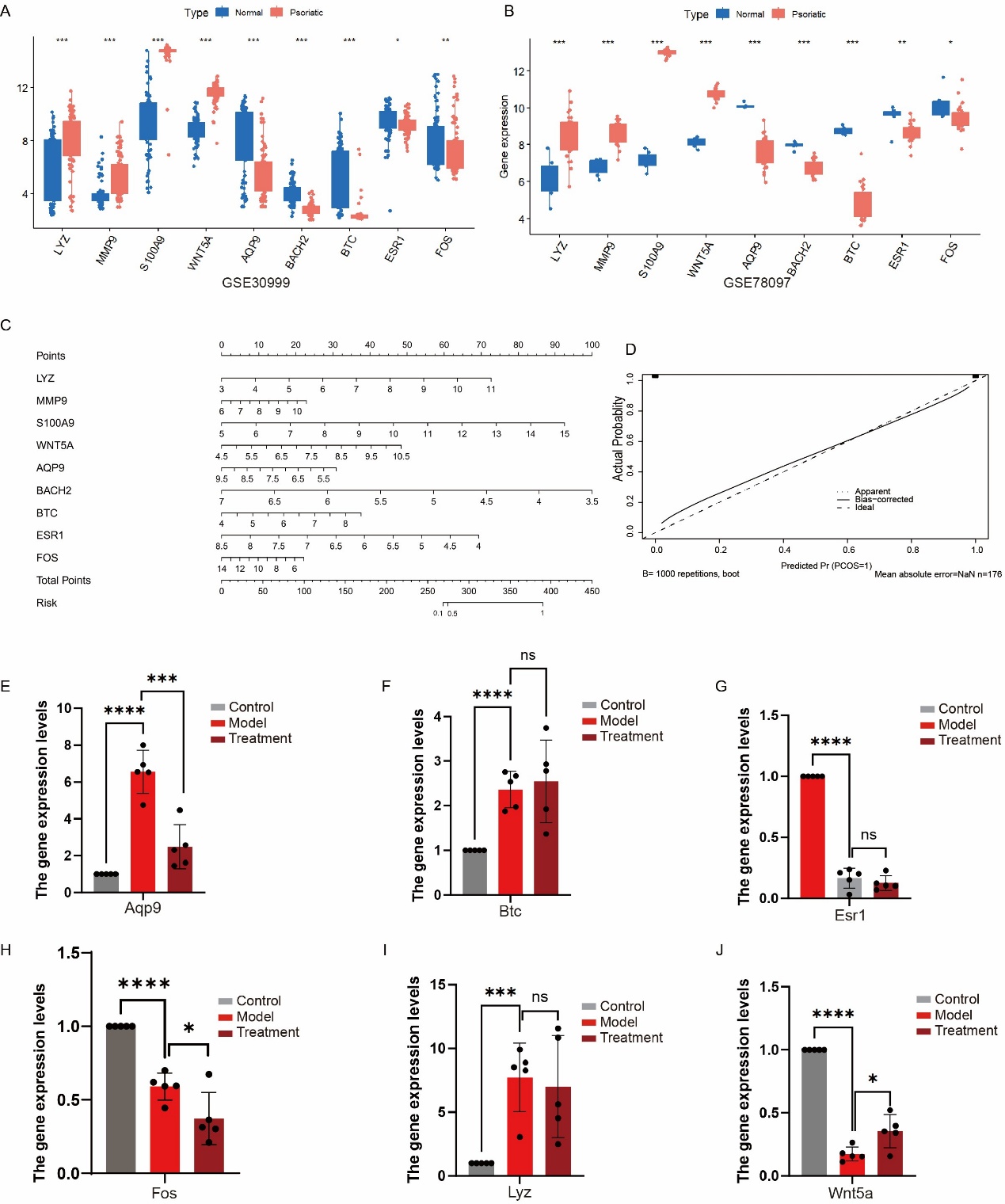
**

**Supplementary Fig 3.** (A) Relative expression levels of hub PIRGs from the GSE30999 datasets. (B) Relative expression levels of hub PIRGs from the GSE78097 datasets. (C) A nomogram predicting the risk of patients with psoriasis. (D) The calibration curves for the nomogram. (E-J) Quantitative real-time PCR analysis of mRNA levels of Aqp9 (E), Btc (F), Esr1 (G), Fos (H), Lyz (I) and Wnt5a (J), with statistical analysis presented. (n = 5). Each experiment was repeated at least three times. ns not significant, *P< 0.05, **P < 0.01, ***P < 0.001, ****P < 0.0001.


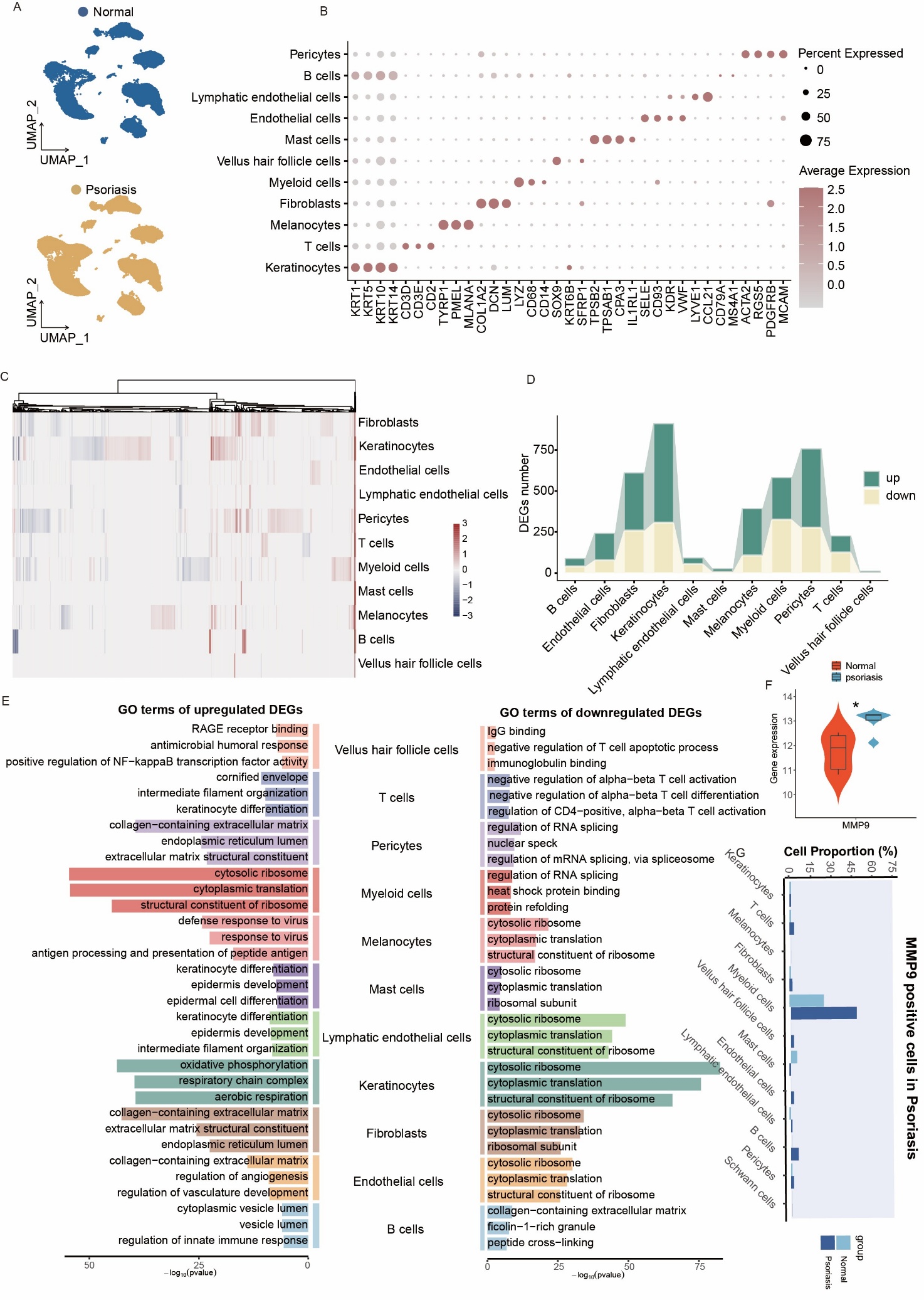


**Supplementary Fig 4.** (A) UMAP plot indicating the patient group for each single cell. (B) Bubble diagram showing characteristic genes in different clusters. (C) Heatmap showing the DEGs (|logFC| > 0.25, adjusted P value < 0.05) during aging across different cell types. (D) Bar plot showing the numbers of DEGs across different cell types. (E) Diagram showing the enrichment of GO terms of cell types with DEGs. (F) The violin plot illustrates the expression levels of S100A9 in myeloid cells under both normal conditions and in psoriasis. ns not significant. (G) Verify the proportion of MMP9-positive cells in different cell types of the validation set, *P< 0.05, **P < 0.01, ***P < 0.001, ****P < 0.0001.

**
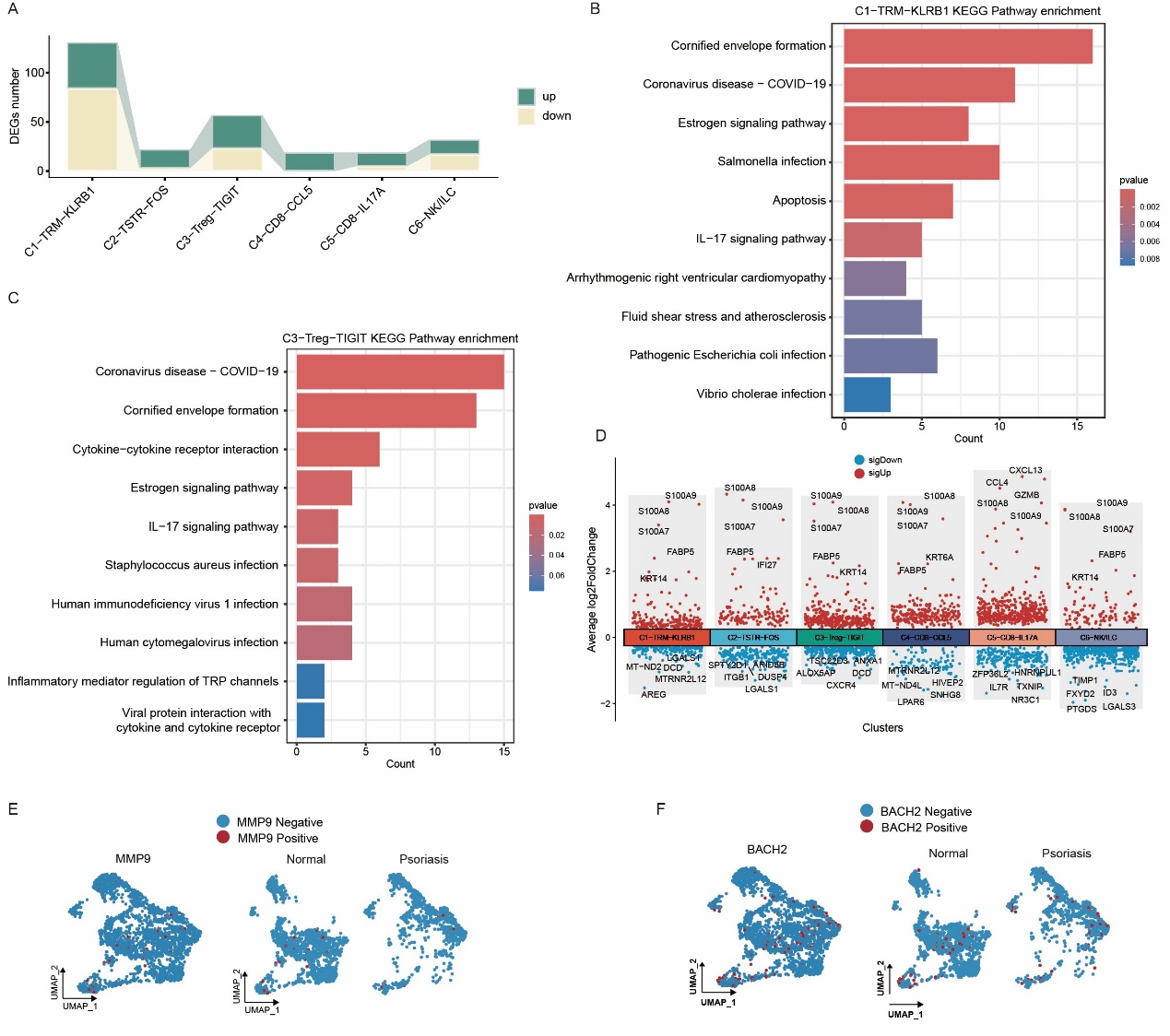
**

**Supplementary Fig 5.** (A) Bar plot showing the numbers of DEGs across different cell types in T cell subclusters. (B-C) Bar plot showing the enrichment of KEGG terms or pathways of C1-TRM-KLRBQ (B) and C3-Treg-TIGIT (C). (D) The dot plot illustrates the differential gene expression across T cell subclusters. (E) UMAP plots showed the total distribution of MMP9 positive T cells (left) and their distribution in normal versus psoriasis (right). (F) UMAP plots showed the total distribution of BACH2 positive T cells (left) and their distribution in normal versus psoriasis (right).

**
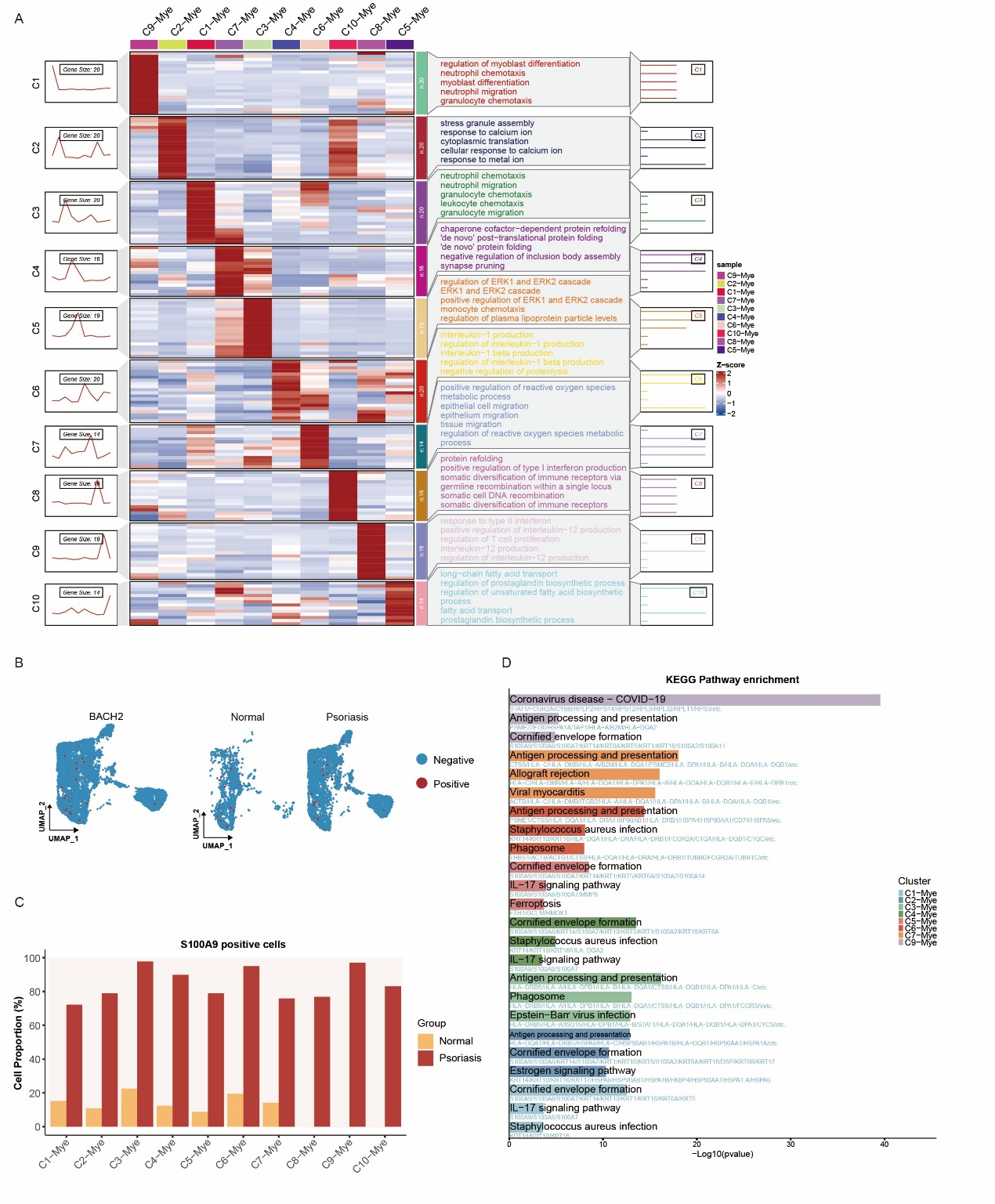
**

**Supplementary Fig 6.** (A) Heatmap showing gene expression signatures of each cell type. Enriched GO terms for each cell type are to the right. (B) UMAP plots showed the total distribution of BACH2 positive myeloid cells (left) and their distribution in normal versus psoriasis (right). (C) Bar plots showing the proportions of and S100A9 positive cells across different myeloid cells types. (D) Diagram showing the enrichment of KEGG terms of T cell types with DEGs of MMP9 positive and MMP9 negative cells.

**
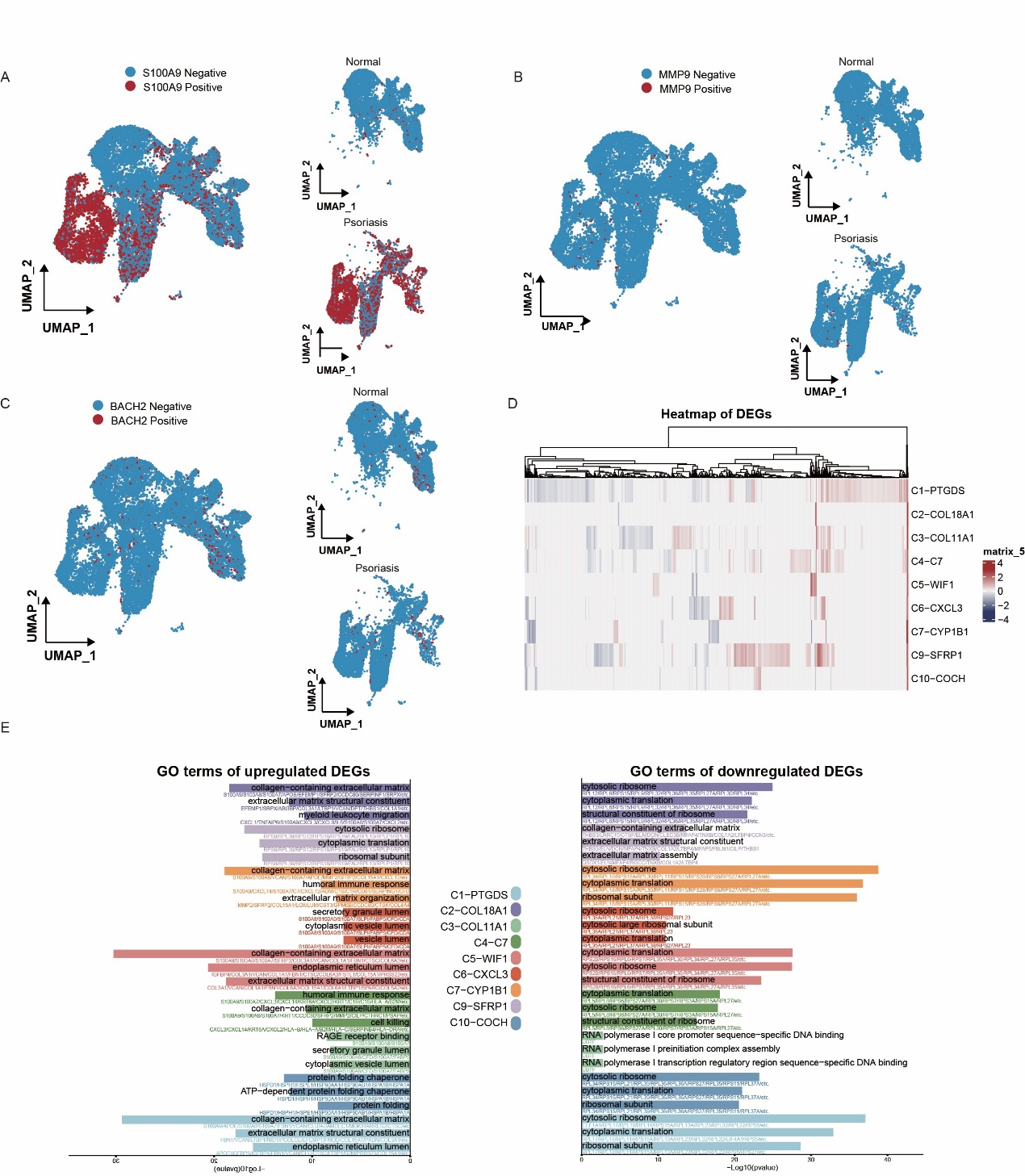
**

**Supplementary Fig 7.** (A) UMAP plots showed the total distribution of MMP9 positive fibroblast (left) and their distribution in normal versus psoriasis (right). (B) UMAP plots showed the total distribution of MMP9 positive fibroblast (left) and their distribution in normal versus psoriasis (right). (C) UMAP plots showed the total distribution of BACH2 positive fibroblast (left) and their distribution in normal versus psoriasis (right). (D) Heatmap showing the DEGs (|logFC| > 0.25, adjusted P value < 0.05) during aging across different fibroblast types. (E) Diagram showing the enrichment of GO terms of cell types with DEGs.

**
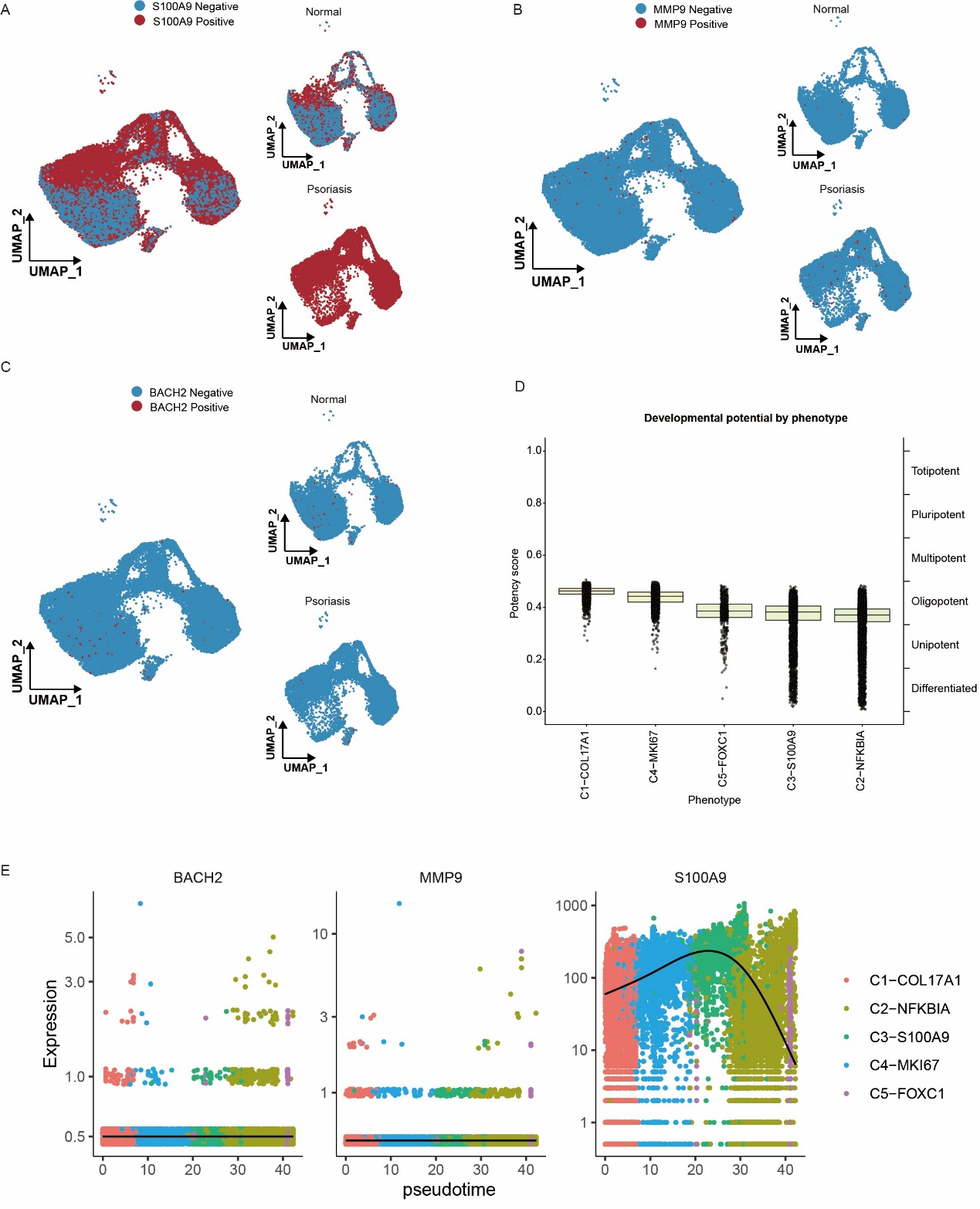
**

**Supplementary Fig 8.** (A) UMAP plots showed the total distribution of MMP9 positive keratinocyte (left) and their distribution in normal versus psoriasis (right). (B) UMAP plots showed the total distribution of MMP9 positive keratinocyte (left) and their distribution in normal versus psoriasis (right). (C) UMAP plots showed the total distribution of BACH2 positive keratinocyte (left) and their distribution in normal versus psoriasis (right). (D) Boxplots showing CytoTRACE research of the developmental potential of keratinocyte subpopulations. (E) Expression dynamics of BACH2, MMP9, and S100A9 along the pseudotime trajectory.

## Supplementary Table

| **Gene** | **Forward primer(5‘-3‘)** | **Reverse primer(5‘-3‘)** |
| --- | --- | --- |
| **β-Actin -** **Mouse** | CCTCTATGCCAACACAGTGC | ACATCTGCTGGAAGGTGGAC |
| **Lyz1-** **Mouse** | GAGACCGAAGCACCGACTATG | CGGTTTTGACATTGTGTTCGC |
| **Mmp9 -** **Mouse** | CTGGACAGCCAGACACTAAAG | CTCGCGGCAAGTCTTCAGAG |
| **S100a9 -** **Mouse** | CCAACATCTGTGACTCTTTAGCC | GCTCAGCTGATTGTCCTGGT |
| **Wnt5a -** **Mouse** | GGAACGAATCCACGCTAAGGGT | AGCACGTCTTGAGGCTACAGGA |
| **Esr1 -** **Mouse** | TCTGCCAAGGAGACTCGCTACT | GGTGCATTGGTTTGTAGCTGGAC |
| **Fos -** **Mouse** | GGGAATGGTGAAGACCGTGTCA | GCAGCCATCTTATTCCGTTCCC |
| **Bach2 -** **Mouse** | GTCGAAAGAGGAAGCTGGACTG | GAGGCAGGAAAAGTTGTCCAGG |
| **Aqp9 -** **Mouse** | TTGCAACGGCAGTTGTGATG | CAAAAGACACCGCTGGGTTG |
| **Btc -** **Mouse** | TTCGTGGTGGACGAGCAAACTC | CCATGACCACTATCAAGCAGACC |
| **β-ACTIN -** **Human** | GAGCACAGAGCCTCGCCTTT | ATCCTTCTGACCCATGCCCA |
| **S100A9 -** **Human** | GCACCCAGACACCCTGAACCA | TGTGTCCAGGTCCTCCATGATG |
